# Recent advances in the asymmetric phosphoric acid-catalyzed synthesis of axially chiral compounds

**DOI:** 10.3762/bjoc.17.185

**Published:** 2021-11-15

**Authors:** Alemayehu Gashaw Woldegiorgis, Xufeng Lin

**Affiliations:** 1 Department of Chemistry, Zhejiang University, Hangzhou, Zhejiang 310027, China

**Keywords:** allenes, atropisomerism, axial chirality, chiral phosphoric acid, heterobiaryls, spiranes

## Abstract

In recent years, the synthesis of axially chiral compounds has received considerable attention due to their extensive application as biologically active compounds in medicinal chemistry and as chiral ligands in asymmetric catalysis. Chiral phosphoric acids are recognized as efficient organocatalysts for a variety of enantioselective transformations. In this review, we summarize the recent development of chiral phosphoric acid-catalyzed synthesis of a wide range of axially chiral biaryls, heterobiaryls, vinylarenes, *N*-arylamines, spiranes, and allenes with high efficiency and excellent stereoselectivity.

## Introduction

Axial chirality is one of the most important properties of nature, resulting from the nonplanar arrangement of four groups in pairs about a chirality axis. These include atropisomerism [1], chiral allenes, spiranes, spiroindanes, and so on [2–3]. Recently, there emerged an enormous demand for enantiopure compounds, not only for pharmaceutical and fine chemical industries, but also for fragrance, flavor, agrochemical, and food industries [4]. Consequently, the importance of axially chiral compounds has been widely recognized in both academic and industrial chemical societies [5]. Therefore, asymmetric synthesis of axial chiral compounds has been paid much attention [6], and great progress has been made in recent years. For example, many remarkable activities have been undertaken to develop strategies such as dynamic kinetic resolution, atroposelective coupling, cycloaddition, and chirality conversion for the construction of axial chirality [7–14].

**Figure 1:**
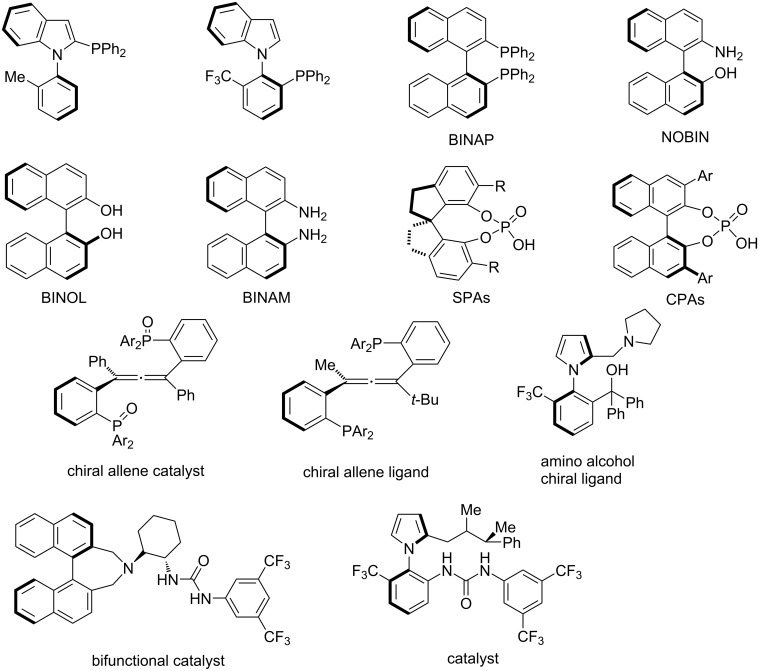
Representative examples of axially chiral biaryls, heterobiaryls, spiranes and allenes as ligands and catalysts.

**Figure 2:**
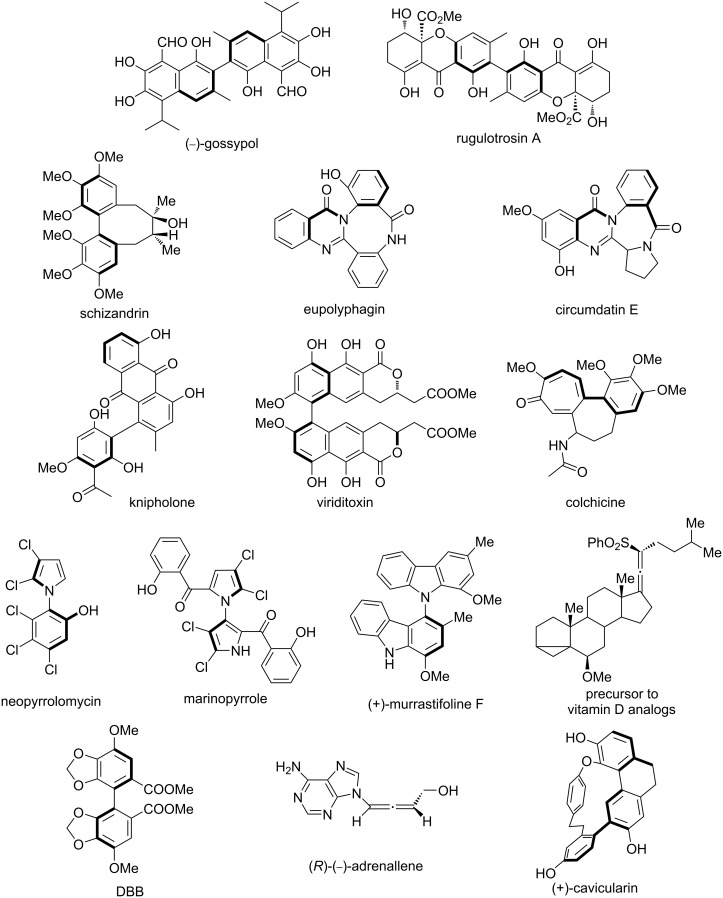
Selected examples of axially chiral drugs and bioactive molecules.

Axially chiral biaryl and heterobiaryl units are widely used as basic building blocks for chiral ligands [14], chiral catalysts [14–17] (Figure 1), various natural products, drugs and bioactive molecules [18–19], pharmaceutical agents [20–21] (Figure 2), and chiral building blocks in modern organic synthesis [5]. During the past decades, both *C*_2_ and non-*C*_2_ symmetric axially chiral biaryl compounds such as BINAP, BINAM, NOBIN and their derivatives BINOL have played a crucial role as ligands in the development of transition-metal-catalyzed enantioselective transformations [9,14,22–23].

**Figure 3:**
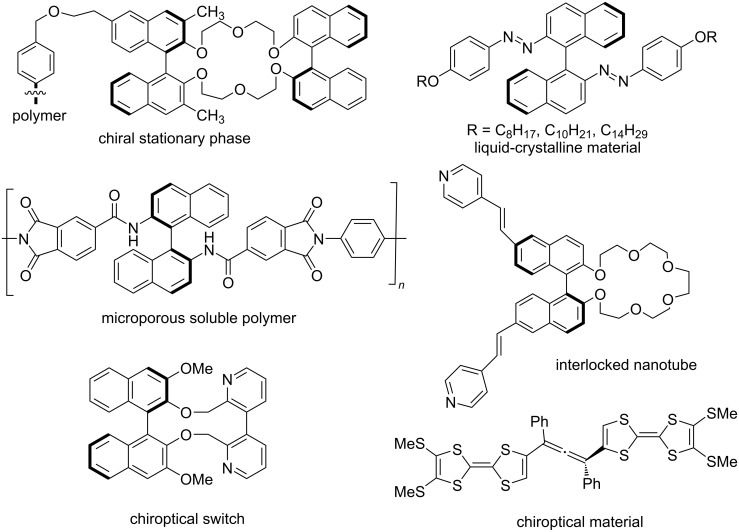
Axially chiral functional materials and supramolecules.

Axial chirality is also found in chiral stationary phases for enantioselective separation, dopants in liquid-crystalline materials, chiroptical molecular switches, microporous soluble polymers, and interlocked nanotubes (Figure 3) [24]. In addition, axially chiral allenes and spiranes [25] are well-known scaffolds widely used in natural products, ligands, organocatalysts, and functional materials as well as versatile chiral building blocks in organic synthesis [14,26–27].

**Figure 4:**
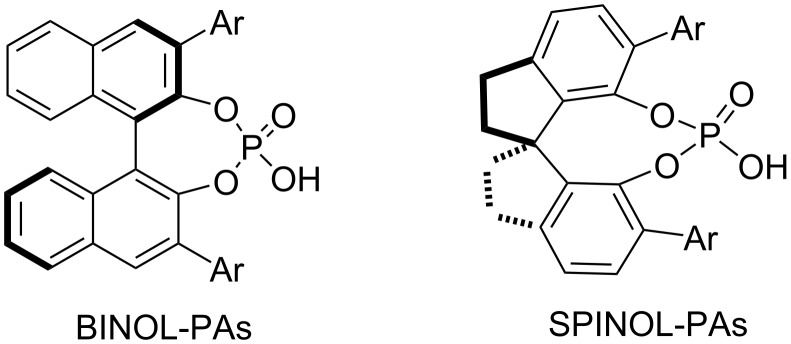
Important chiral phosphoric acid scaffolds used in this review.

Chiral phosphoric acids represent an important and widely used class of catalysts for a variety of enantioselective transformations, especially for carbon–carbon and carbon–heteroatom bond-forming reactions [15,28]. They are important for the development of axially chiral compounds, which are involved in the design of chiral catalysts and ligands. Currently, chiral phosphoric acids are widely used in stereoselective oxidative/cross-coupling of two aryl counterparts, asymmetric control of aromatic ring formation, atroposelective functionalization of biaryl compounds, and so on [17,29–30]. In this context, Akiyama (2004) described that chiral phosphoric acids (CPAs) have great potential for catalysis of a wide range of reactions to achieve good to perfect enantioselectivities. This is due to their ability to act as synergistic bifunctional catalysts bearing both Brønsted acidic and Lewis basic sites, with the 3,3′-substituents playing a crucial role in achieving excellent enantioselectivity [31]. The widespread use of phosphoric acids and phosphates as chiral acids, chiral anions, and ligands is one of the most important achievements of modern enantioselective catalysis. The atropochiral BINOL, H_8_-BINOL and SPINOL [32] derived phosphoric acids (Figure 4) [33–34] play a crucial role in asymmetric catalysis, the construction of numerous axially chiral biaryls/heterobiaryls [21,35–38], and other useful asymmetric transformations [5].

Although chiral phosphoric acids have shown promising properties in asymmetric catalysis and play a significant catalytic role in the development of axially chiral compounds with biaryls, heterobiaryls, atropisomeric arylalkenes, allenes, and spiranes, there are only few comprehensive reviews in this area. Therefore, the aim of this review is to provide readers with an overview of recent advances in the asymmetric phosphoric acid-catalyzed synthesis of axially chiral compounds and to suggest that much more attention should be paid to these catalysts in order to promote asymmetric synthesis.

## Review

### 1. Enantioselective synthesis of atropisomeric biaryls

**Scheme 1:**
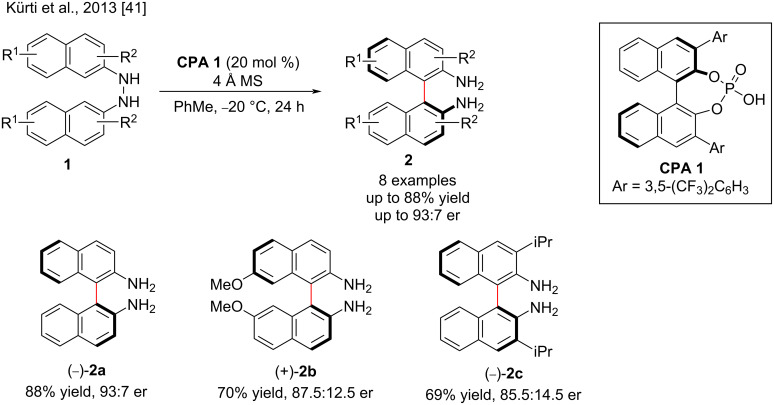
Atroposelective aryl–aryl-bond formation by employing a facile [3,3]-sigmatropic rearrangement.

Direct C–H functionalization strategies for the atroposelective aryl–aryl cross-coupling using various transition-metal catalysts has rarely been successful for the enantioselective construction of hindered biaryls [39] due to the discord between the temperature tolerance of the rotational axis and the high temperature required for C–H activation and suffered from poor chemoselectivity [40]. However, the realization of this redox-neutral aryl–aryl cross-coupling is a formidable challenge. Therefore, the discovery of efficient catalysts and ligands to achieve high stereoselectivity is a fundamental issue in catalytic asymmetric synthesis [14]. In this section, we will present pioneering examples of chiral phosphoric acid-catalyzed asymmetric syntheses of axially chiral biaryls. In 2013, Kürti and co-workers reported a chiral phosphoric acid-catalyzed aryl–aryl-bond formation process for the regio- and atroposelective synthesis of 2,2′-diamino-1,1′-binaphthalenes (BINAMs) from achiral *N*,*N*′-binaphthylhydrazines (Scheme 1). In the presence of chiral phosphoric acids (**CPA 1**), the reaction undergoes a simple [3,3]-sigmatropic rearrangement, giving the corresponding products **2** in good yield (up to 88%) and enantioselectivity (up to 93:7 er). The density functional calculations showed that the chiral phosphoric acid proton forms an H-bond with nitrogen atoms of **1** and the phosphate acts as a chiral counterion, resulting in a [3,3]-sigmatropic rearrangement with controlled stereoselectivity [14,41].

**Scheme 2:**
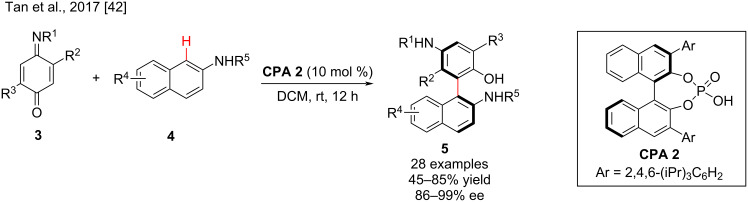
Atroposelective synthesis of axially chiral biaryl amino alcohols **5**.

In 2017, Tan and co-workers developed an organocatalytic atroposelective synthesis of axially chiral biarylamino alcohols from the reaction of quinone ester (**3**) and various 2-naphthylamines **4**. In the presence of **CPA 2**, the axially chiral biarylamino alcohols **5** were obtained in moderate to good yields (up to 85%) and high to excellent enantioselectivities (up to 99% ee) (Scheme 2). The electronic properties of the substituents on the 2-naphthylamine showed remarkable effects on the chemical yield but had negligible impact on the enantioselectivity [42].

**Scheme 3:**
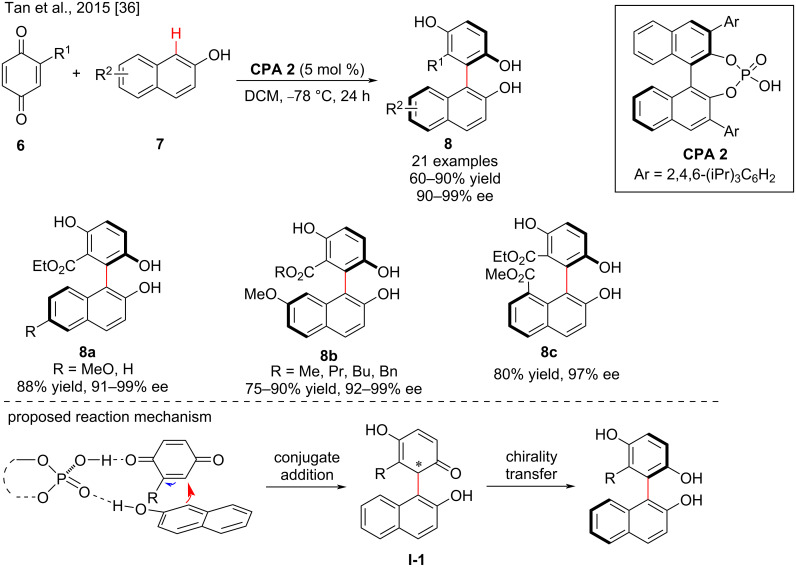
The enantioselective reaction of quinone and 2-naphthol derivatives.

The direct arylation reaction of quinones **6** and 2-naphthols **7** was described by Tan and co-workers in 2015. The corresponding axially chiral biaryl diols **8** were prepared in good yields (60–90%) and excellent enantioselectivities (90–99% ee) in the presence of **CPA 2** (Scheme 3). The stereoselectivity of the reaction is only moderately affected by the position and electronic properties of the substituents on the aromatic ring [36]. Experiments showed that chiral phosphoric acid **CPA 2** acted as a bifunctional organocatalyst, that activates 2-naphthols and quinone derivatives via multiple H-bonds and promotes the first step of the enantioselective conjugative addition to generate intermediate **I-1** and transfers the its central chirality information to the axial chirality to give the chiral biaryldiols (Scheme 3) [14].

**Scheme 4:**
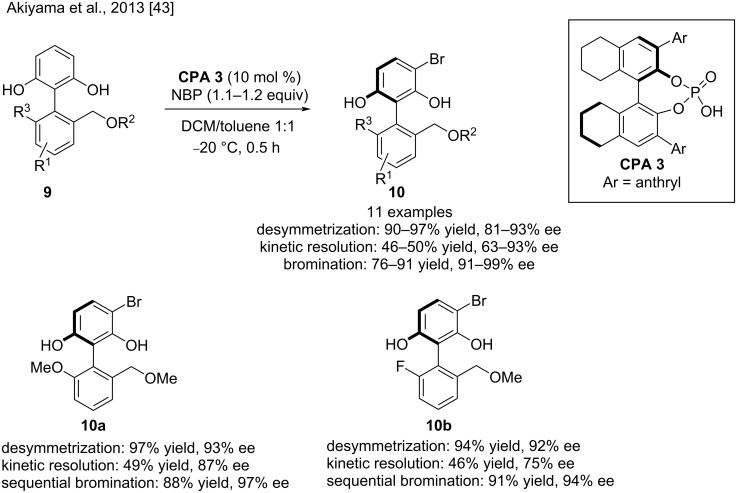
Enantioselective synthesis of multisubstituted biaryls.

In 2013, Akiyama and co-workers described the enantioselective preparation of multisubstituted biaryls by the desymmetrization strategy, which was further enhanced by the subsequent asymmetric reaction (kinetic resolution) in the presence of chiral phosphoric acid **CPA 3**. In this work, various EWG- and EDG-containing substrates were incorporated, and chiral biaryls **10** were obtained with good to excellent selectivities of 81–93% ee by desymmetrization and 63–96% ee by kinetic resolution. The subsequent asymmetric bromination reaction afforded monobrominated biaryls with excellent enantioselectivities up to 99% ee (Scheme 4). The experimental and computational studies showed that the highly organized hydrogen-bonding network between a substrate, a chiral phosphoric acid catalyst (**CPA 3**), and a brominating reagent (*N*-bromophthalimide) plays a crucial role in achieving the excellent selectivities [43].

**Scheme 5:**
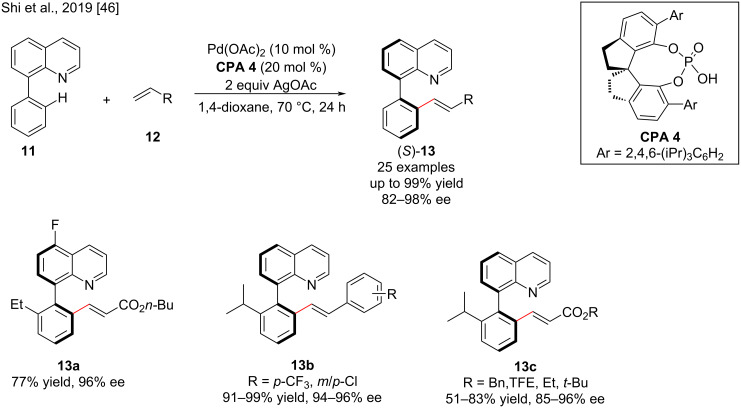
Enantioselective synthesis of axially chiral quinoline-derived biaryl atropisomers mediated by chiral spirophosphoric acid catalyst **CPA 4**.

The axially chiral biaryl-2-amines, N-heteroarenes [44] and derivatives are ubiquitous structural motifs and have profound applications in biochemistry and asymmetric catalysis [45]. These chiral biaryls can be prepared by asymmetric C–H activation. The C–H activation or functionalization can be achieved by a metal-catalyzed chiral phosphoric acid ligand-assisted method, which offers distinct possibilities to provide various chiral biaryl compounds by changing different directing groups (DGs) [46]. Since the important reports by Akiyama and Terada [31,47], chiral phosphoric acids have received much attention in the efficient construction of chiral molecules not only by using them as organocatalysts, but also by applying them as ligands in transition-metal catalysis due to their multifunctionality (Brønsted acidity/basicity, hydrogen-bonding units, and counter-anions toward metals) [48–49]. In 2019, Shi, Lin, and co-workers achieved an enantioselective synthesis of axially chiral quinoline-derived biaryl atropisomers via Pd-catalyzed C–H olefination of 8-phenylquinoline (**11**) using a novel chiral spirophosphoric acid (**CPA 4**). A wide range of quinoline-derived biaryls **13** with various substituents was synthesized in good to excellent yields (up to 99%) with excellent enantioselectivities (up to 98% ee) (Scheme 5). A working model for the origin of enantioselectivity in C–H olefination was provided by density functional theory calculations [46].

**Scheme 6:**
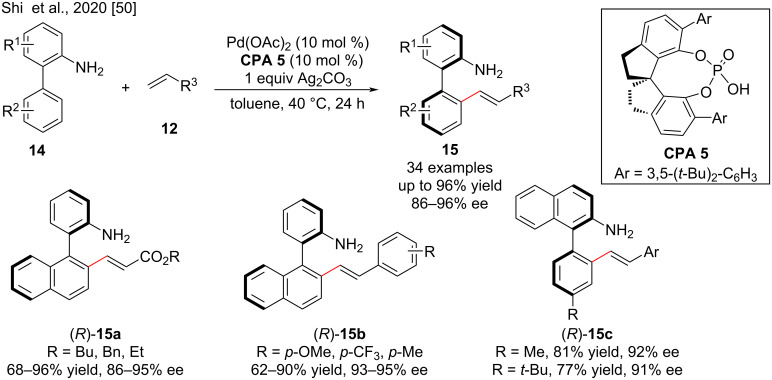
Pd-Catalyzed atroposelective C–H olefination of biarylamines.

Recently, Shi, Lin and co-workers reported the atroposelective synthesis of axially chiral biaryls by Pd(II)-catalyzed free amine-directed atroposelective C–H olefination using chiral spirophosphoric acid **CPA 5** as an efficient ligand and Ag_2_CO_3_ as the oxidant. The C–H olefination of various biaryl-2-amines with acrylates and styrenes was carried out and afforded the desired products **15** in good yields (up to 96% yield) with high enantioselectivities (up to 96% ee). The electronic properties of the side chains of the biaryl compounds have a significant effect on the reactivity, but little on the enantiocontrol. In general, biaryl-2-amines with electron-donating groups showed higher reactivity than those with electron-withdrawing groups (Scheme 6). **CPA 5** activates the reaction by creating a rigid and narrow chiral pocket, thus leading to better noncovalent interactions with the substrates. Moreover, the SPA ligand loading of 1 mol % is effective for this reaction under mild conditions [50].

**Scheme 7:**
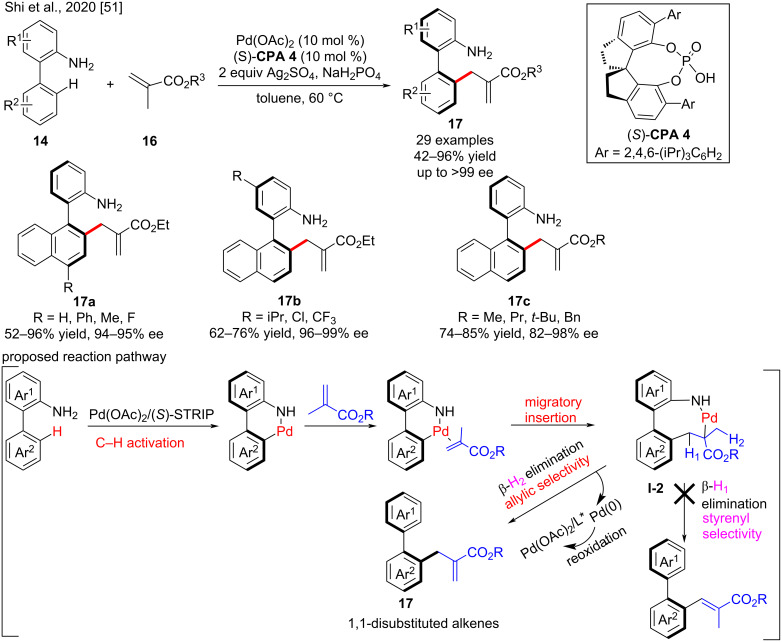
Palladium-catalyzed directed atroposelective C–H allylation.

Moreover, Shi, Lin and co-workers reported the first Pd(II)-catalyzed directed atroposelective C–H allylation of 2-(naphthalen-1-yl)aniline derivatives **14** with methacrylates **16** via β-H elimination using (*S*)-**CPA 4** as a ligand (Scheme 7) [51]. The directing group (NH_2_) facilitated the C–H activation on the other aromatic ring and ensured its coordination with Pd to form palladacycle **I-2** to restrict bond rotations and promote exclusive allylic selectivity via β-H^1^ elimination as opposed to styrenyl selectivity. In addition, the methacrylates were used as allyl surrogates to overcome the reactivity problem caused by steric hindrance of 1,1,-disubstituted alkenes, and the electron-withdrawing esters enhance the migratory insertion to form palladacycle **I-2**. The CPA generates the chiral environment and played a crucial role in favoring of atropisomerism. A series of axially chiral biaryl-2-amines with 1,1-disubstituted alkenes **17** was efficiently synthesized in moderate to excellent yields (42–96%) with excellent enantioselectivities (up to >99% ee).

### 2. Enantioselective synthesis of atropisomeric heterobiaryls

#### 2.1. Synthesis of atropisomeric arylindoles

**Scheme 8:**
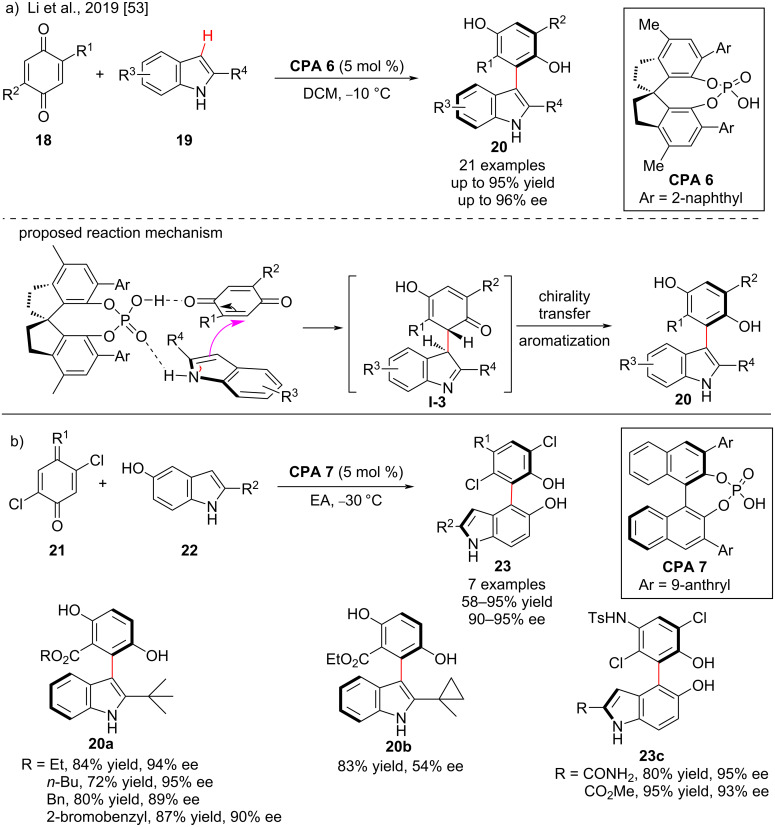
Enantioselective synthesis of axially chiral (a) aryl indoles and (b) biaryldiols.

The indole scaffold is found in many natural products, drugs, and bioactive molecules. Moreover, the introduction of axial chirality into the indole scaffold is receiving attention due to its widespread use [45,52]. In 2019, Li and co-workers developed a synthetic strategy for the atroposelective construction of phenylindole **20** by the chiral phosphoric acid**-**catalyzed cross-coupling of quinones **18** and indoles **19**. In this reaction, the chiral phosphoric acid (*R*)-**CPA 6** acts as a bifunctional catalyst to activate indoles and quinones through a dual H-bond activation mode to form the intermediate **I-3**, and aromatization with central-to-axial chirality transfer occurs to afford the axially chiral phenylindoles in good yield (76–92%) with good to excellent enantioselectivity (88–96% ee, Scheme 8a). The position and electronic properties of the substituents on the aromatic rings of the indole have limited influence on the reactivity and remarkable effects on the enantioselectivity. For example, in the case of indole **20b**, the enantioselectivity was drastically reduced due to steric hindrance present at the 2-position of the indole, which plays a crucial role in the stability of axial chirality.

In 2019, Li and co-workers further investigated the feasibility of 5-hydroxyindoles **22** with iminoquinones **23** under optimized reaction conditions. In the presence of 5 mol % (*R*)-**CPA 7**, the desired biaryldiol products **23** were obtained in moderate to good yields (58–95%) with excellent enantioselectivity (90–95% ee, Scheme 8b) [53]. The synthesized axially chiral phenylindoles have the potential to be used as chiral ligands in asymmetric catalysis [54] and are ubiquitous in natural products with considerable bioactivity [55–56].

**Scheme 9:**
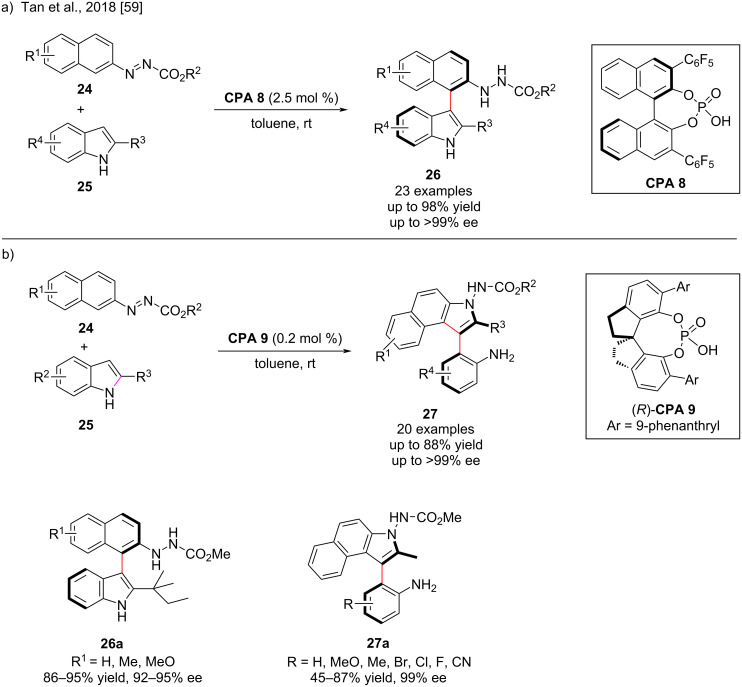
Asymmetric arylation of indoles enabled by azo groups.

**Scheme 10:**
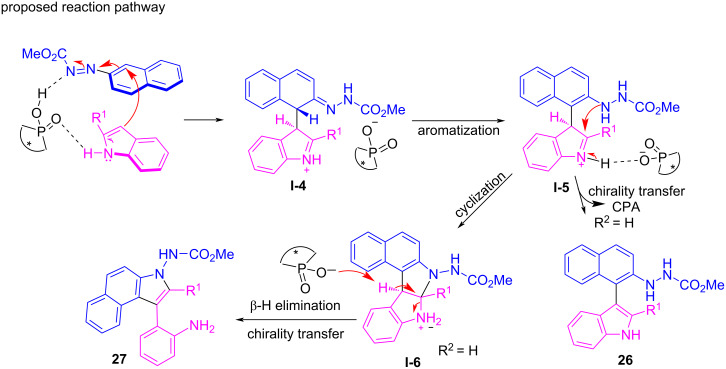
Proposed mechanism for the asymmetric arylation of indoles.

Organocatalytic aryl C–H activation via a nonradical process represents an enormous challenge in organic synthesis, although the nucleophilic aromatic substitution with cleavage of the electrophilic aryl C–H bond has only recently been developed by transition-metal-catalyzed aryl C–H activation [57]. In the presence of a chiral phosphoric acid, the azo group has recently been revealed to be a useful moiety that may efficiently activate an aromatic ring for formal nucleophilic aromatic substitution, resulting in the cleavage of the aryl C–H bond and direct arylation of the nucleophile [58]. In 2018, Tan and co-workers showed that azo groups enable the organocatalytic asymmetric arylation of indoles. The nucleophilic aromatic substitution between the azobenzene derivative **24** and indoles **25** was carried out in the presence of 2.5 mol % chiral phosphoric acid (**CPA 8**, Scheme 9a), leading to the intermediate **I-4**, which was subsequently aromatized to give the intermediate **I-5** (Scheme 10). The axially chiral arylindole **26** was synthesized from intermediate **I-5** via chirality transfer with high isolated yield (up to 98%) and excellent enantioselectivity (up to >99% ee, Scheme 9a) [59]. The authors also reported that the reaction of 2-substituted indoles with azobenzenes proceeded smoothly in the presence of the chiral phosphoric acid catalyst (*R*)-**CPA 9**. At a catalyst loading of 0.2 mol %, the intermediate **I-5** was simultaneously cyclized to **I-6**, followed by β-H elimination and chirality transfer to afford another type of axially chiral indoles **27** (Scheme 10) bearing aniline groups by direct arylation and rearrangement in moderate to good yields with excellent enantioselectivities of mostly 99% ee (Scheme 9b) [59]. The electronic properties and position of the substituents on the azobenzene ring did not affect the yields, whereas the electronic properties of the substituents on the indole ring did.

**Scheme 11:**
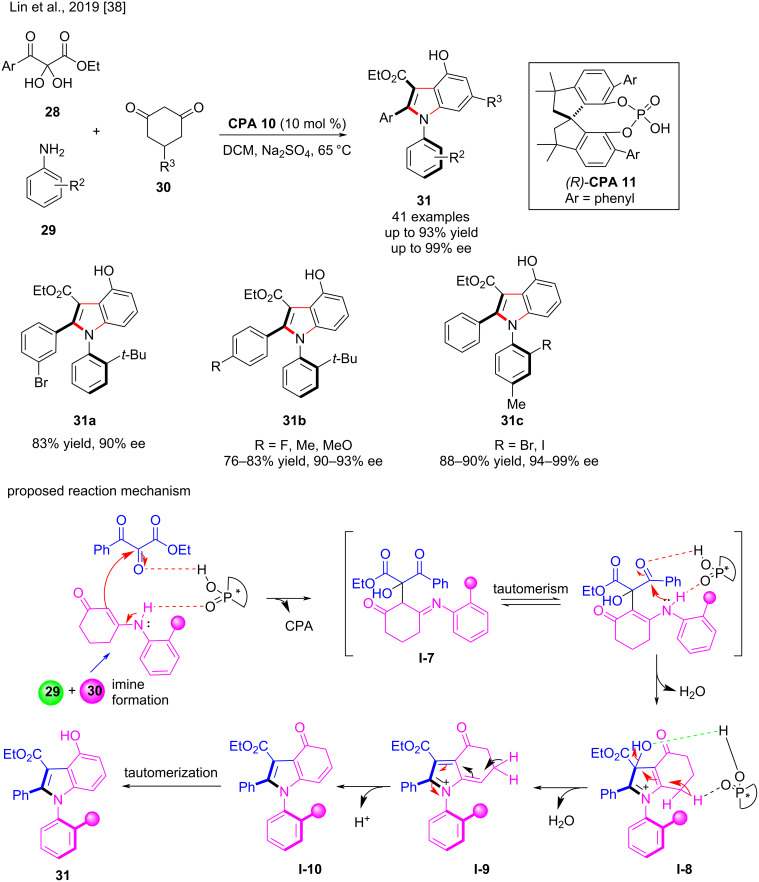
Enantioselective synthesis of axially chiral *N*-arylindoles [38].

In 2019, Lin and co-workers reported the asymmetric three-component cascade reaction of 2,3-diketo esters **28**, aromatic amines **29**, and 1,3-cyclohexanediones **30** to prepare axially chiral arylindoles **31** in a highly enantioselective manner. A wide range of substrates was subjected to the reaction in the presence of the chiral phosphoric acid **CPA 9** to afford axially chiral *N*-arylindoles **31** in good yields (up to 93%) with good to exceptional enantioselectivities (up to 99% ee, Scheme 11). The chiral spirocyclic phosphoric acid catalyst developed by our group is critical for increasing the enantioselectivity in this cascade reaction [38]. This catalyst can facilitate the aldol reaction to generate a stereocenter (**I-7**), which can then be converted to axial chirality (**I-8** to **I-10**) and finally aromatized to give **31** (Scheme 11) [60].

**Scheme 12:**
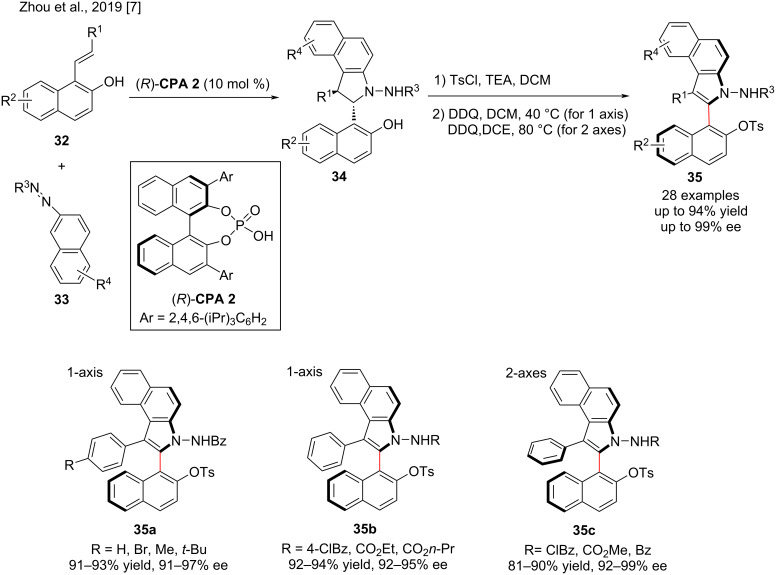
Enantioselective [3 + 2] formal cycloaddition and central-to-axial chirality conversion.

Zhou and co-workers published an excellent paper in 2019 on the conversion of central to axial chirality in an enantioselective [3 + 2] annulation of 1-styrylnaphthols **32** with azonaphthalenes **33**. Under defined conditions, the cycloaddition product **34** was prepared in high yield (99%) with exclusive diastereoselectivity and 99% ee in the presence of the chiral phosphoric acid **CPA 2**. Subsequently, using the chiral phosphoric acid-catalyzed [3 + 2] formal cycloaddition and a moderate DDQ oxidation method over **34**, enantiomerically enriched 2,3-diarylbenzoindoles **35** were successfully prepared by performing a central-axial chirality conversion by oxidative aromatization. With excellent diastereoselectivities (all >20:1) and enantioselectivities (87–99% ee), the target products **35** containing different groups were obtained in high yield (up to 94%) (Scheme 12). In this transformation, electron-donating and withdrawing groups were tolerated [7].

**Scheme 13:**
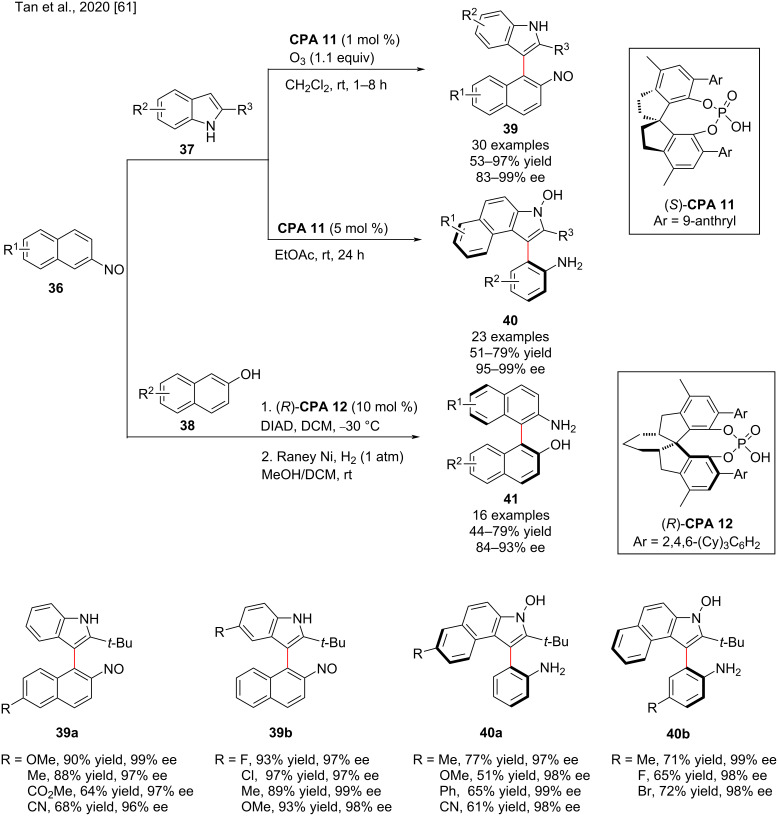
Organocatalytic atroposelective arene functionalization of nitrosonaphthalene with indoles.

**Scheme 14:**
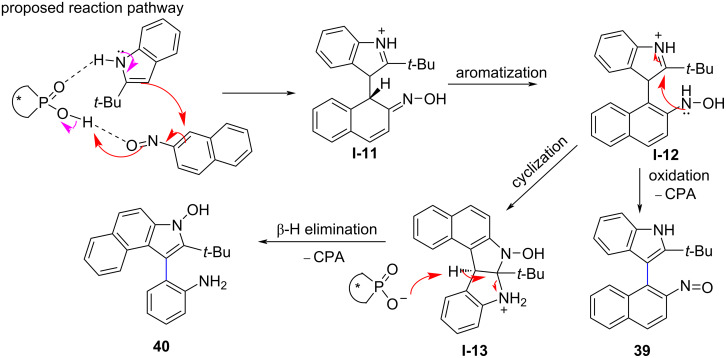
Proposed reaction mechanism for the atroposelective arene functionalization of nitrosonaphthalenes.

In 2020, Tan and co-workers disclosed the phosphoric acid-catalyzed atroposelective arene functionalization of nitrosonaphthalene with indoles to form atropisomeric indole-naphthalenes **39** and indole-anilines **40** by a nucleophilic aromatic substitution reaction [61] (Scheme 13). Various 2-nitrosonaphthalenes **36** and indoles **37** with different substitutions were subjected to nucleophilic aromatic substitution reaction catalyzed by **CPA 11** to form an intermediate **I-11**, which was then re-aromatized to give **I-12**, and then oxidized to give **39**. In addition, the intermediate **I-12** was cyclized to form the intermediate **I-13** followed by β-H elimination to give another axially chiral arylindole framework **40** (Scheme 14). Both products **39** and **40** were obtained in moderate to excellent yields (up to 99%) and good to excellent enantioselectivity (83–99% ee, Scheme 13). Moreover, the reactions provide easy access to the privileged NOBIN (2-amino-2’-hydroxy-1,1’-binaphthyl) structures **41**. The additional insights into the origins of enantiocontrol were described by DFT calculations.

**Scheme 15:**
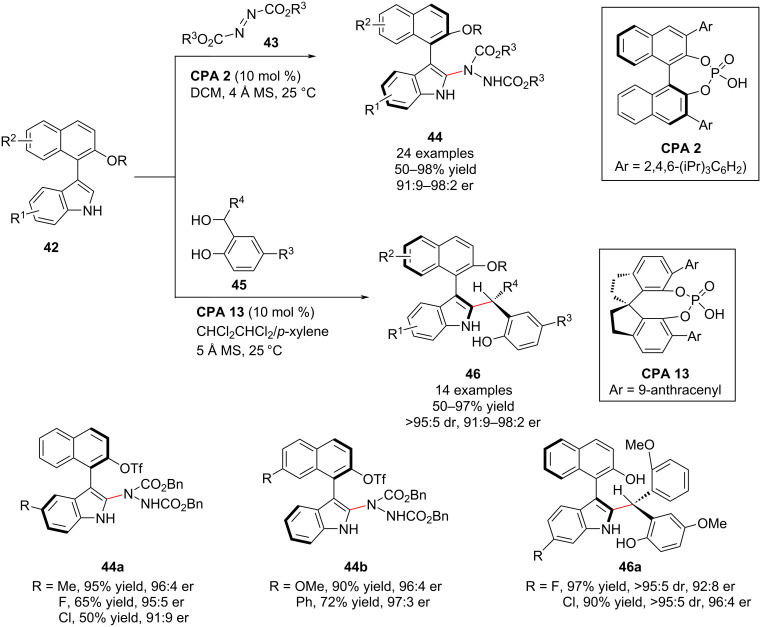
Asymmetric construction of axially chiral naphthylindoles [65].

Owing to the presence of axially chiral indole-based biaryl scaffolds such as naphthylindoles and phenylindoles in bioactive molecules and chiral catalysts [62–64], the construction of this scaffold has recently become valuable and attracted the attention of chemists [2,64]. In this context, Shi and co-workers reported a new strategy for the enantioselective synthesis of axially chiral naphthylindoles via the asymmetric addition reaction of racemic naphthylindole **42** with azodicarboxylate **43** under chiral phosphoric acid catalysis. In the presence of **CPA 2, 42** and **43** reacted and underwent dynamic kinetic resolution to afford naphthylindoles **44** with axial chirality in moderate to good yields (50–98%) and high enantioselectivity (91:9 to 98:2 er, Scheme 15) [65].

In addition, the authors also succeeded in preparing naphthylindoles **46**, which exhibit both axial and central chirality, through the addition reaction of racemic naphthylindoles **42** and *o*-hydroxybenzyl alcohols **45** using chiral phosphoric acid **CPA 13**. This reaction afforded products **46** in moderate to good yields (50–97%), excellent diastereoselectivities (>95:5 dr), and high enantioselectivities (91:9 to 98:2 er, Scheme 15). The control experiments showed that the N–H group in naphthylindoles, the OH group in phenol, and the carboxylate group in azodicarboxylate play a crucial role in both the reactivity and enantioselectivity of the reaction due to the formation of hydrogen bonds with CPA. In addition, the chiral phosphoric acid catalysts generate the chiral environment and showed the pleasing role in favoring atropisomerism [65].

**Scheme 16:**
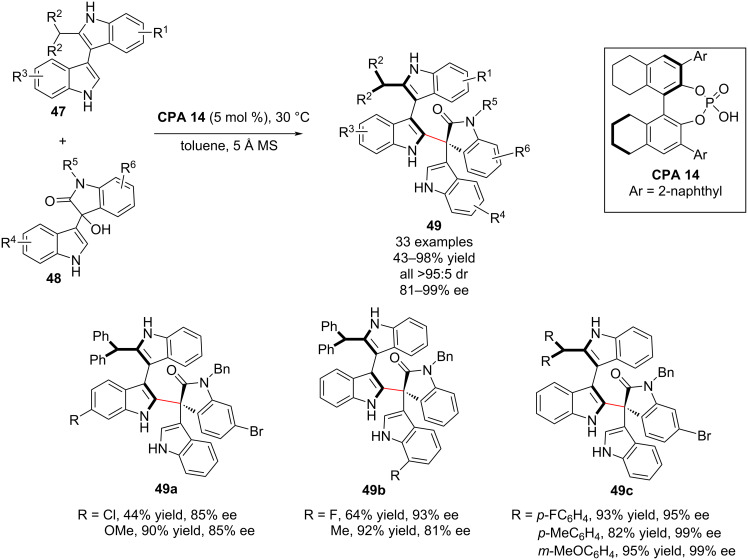
Enantioselective synthesis of axially chiral 3,3’-bisindoles [66].

In 2019, Shi and co-workers also achieved the first catalytic asymmetric construction of axially chiral 3,3’-bisindole scaffolds **49** bearing both axial and central chirality by employing the **CPA-14**-catalyzed asymmetric addition reaction of 2-substituted 3,3’-bisindoles **47** to isatin-derived 3-indolylmethanols **48**. The isatin-derived 3-indolylmethanols and 2-substituted 3,3’-bisindole substrates bearing different substituents afforded the axially chiral 3,3’-bisindole scaffolds **49** in moderate to high yields (up to 98%) and with excellent stereoselectivities (all >95:5 dr, >99% ee, Scheme 16). Moreover, the introduction of a bulky group into the *ortho*-position of prochiral 3,3’-bisindoles allowed the synthesis of new members of axially chiral biaryls (3,3’-bisindole derivatives) bearing both axial and central chirality in high yields and excellent stereoselectivities. Moreover, this methodology represents a new strategy for the catalytic enantioselective synthesis of axially chiral 3,3’-bisindole backbones from prochiral substrates [66].

**Scheme 17:**
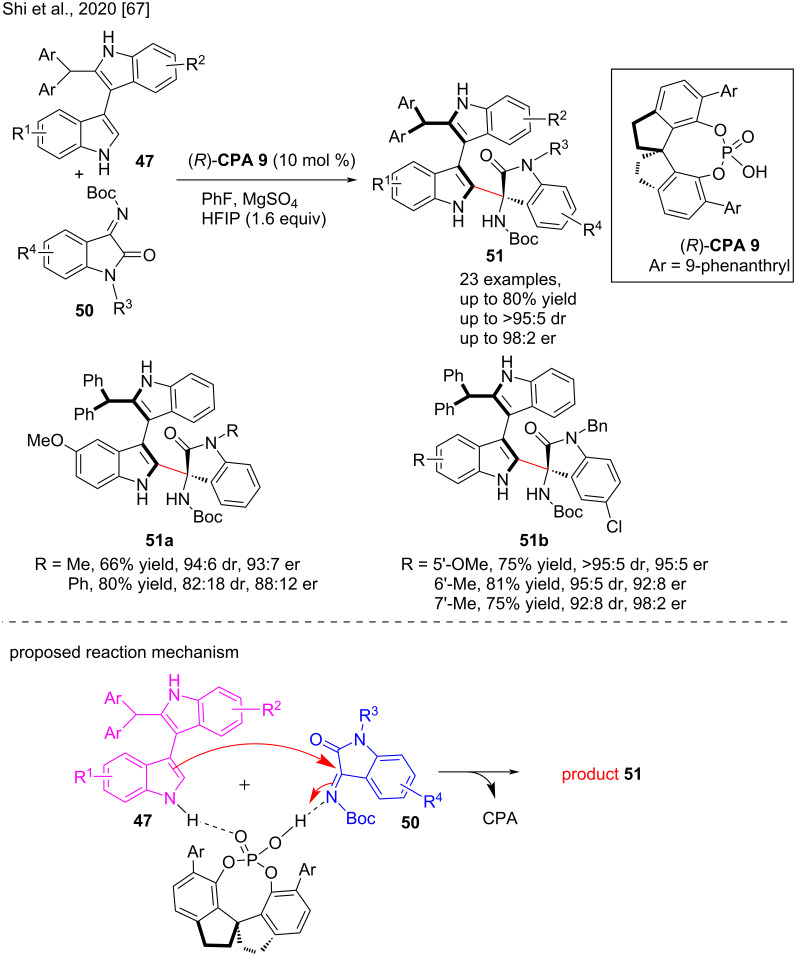
Atroposelective synthesis of 3,3’-bisiindoles bearing axial and central chirality.

The atroposelective synthesis of a new class of 3,3'-bisindoles **51** with axial and central chirality by dynamic kinetic resolution of 2-substituted 3,3'-bisindoles via asymmetric nucleophilic addition reactions using isatin-derived imines **50** as electrophiles was reported by Tan, Shi and co-workers in 2020. Enantioenriched 3,3'-bisindoles with a biologically essential chiral 3-aminooxindole unit can be readily prepared by these methods. **CPA 9** utilizes hydrogen bonding to activate both reactants, resulting in enantioenriched 3,3'-bisindole derivatives with multiple chirality. In the presence of the chiral phosphoric acid catalyst **CPA 9**, various groups of 2-substituted 3,3'-bisindoles and isatin-derived imines were tolerated and gave good to high yields (up to 80%) and moderate to excellent stereoselectivities (up to 98:2 er, >95:5 dr, Scheme 17) [67].

**Scheme 18:**
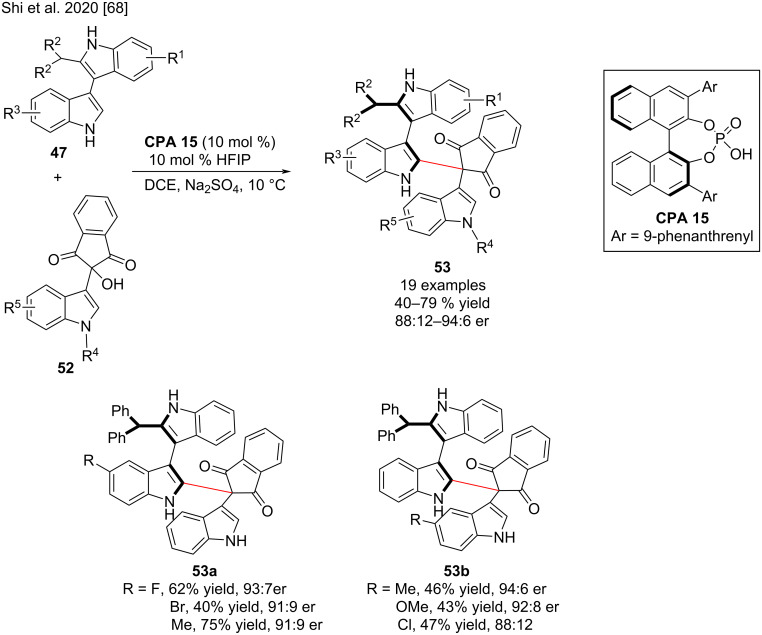
Enantioselective synthesis of axially chiral 3,3’-bisindoles bearing single axial chirality.

In 2020, Shi and co-workers reported the chiral phosphoric acid (CPA)-catalyzed enantioselective addition reaction of 3,3'-bisindoles **47** and ninhydrin-derived 3-indolylmethanols **52** for the synthesis of 3,3'-bisindoles **53** with single axial chirality. In the presence of **CPA 15**, the axially chiral 3,3′-bisindoles **53** were synthesized via a dynamic kinetic resolution (DKR) process in overall moderate yields (up to 79%) and good enantioselectivities (up to 94:6 er, Scheme 18) [68]. The authors investigated the high rotation barrier and stability of the axially chiral products and found that 3,3'-bisindole scaffolds with a single axial chirality are quite stable because of the bulkiness of the ninhydrin group. They also investigated the rotational barrier of substrate **47** and confirmed that it could be rapidly racemized during the reaction process, suggesting that dynamic kinetic resolution could be used to accomplish enantioselective synthesis of axially chiral 3,3'-bisindoles. The mechanistic studies showed that the N–H group of 3,3′-bisindoles **47** played a crucial role in carrying out the addition reaction with substrates **52** possibly by forming a hydrogen bond with **CPA 15**. Moreover**,** the N–H group of ninhydrin-derived 3-indolylmethanols **52** was crucial in increasing the reactivity and enantioselectivity, but it is not important for ninhydrin-derived substrates to carry out the addition reaction. This suggests that this substrate could produce an ion-pair interaction with **CPA 15** in addition to the hydrogen-bonding interaction.

#### 2.2. Synthesis of miscellaneous atropisomeric heterobiaryls

**Scheme 19:**
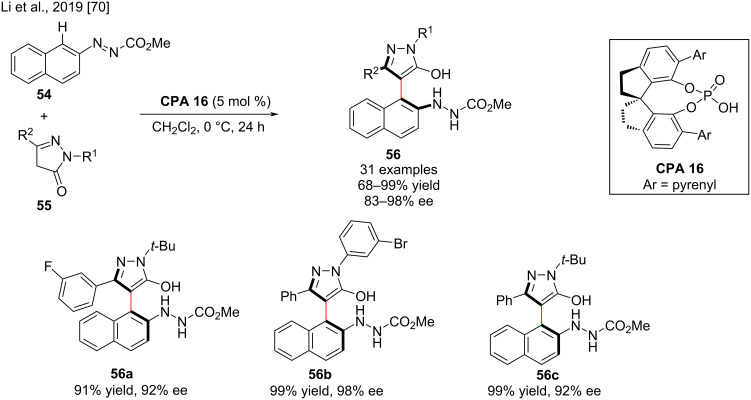
Enantioselective reaction of azonaphthalenes with various pyrazolones.

Axially chiral pyrazole scaffolds are commonly found in natural products and drugs and are often used as valuable building blocks in organic synthesis [69]. Herein, Li and co-workers developed a new asymmetric synthesis of atropisomeric pyrazole derivatives **56** via an enantioselective reaction of azonaphthalene **54** with pyrazolone **55** using 5 mol % chiral phosphoric acid **CPA 16**. Substrates bearing electron-donating and electron-withdrawing groups gave the desired axially chiral pyrazole derivatives **56** in good yields (up to 99%) with excellent enantioselectivities (up to 98% ee, Scheme 19). Density functional theory calculations revealed that the chiral phosphoric acid acts as a proton-transfer shuttle to assist proton transfer from the OH group of pyrazolone-enol intermediates to the N–N double bonds of azonaphthalenes [70].

**Scheme 20:**
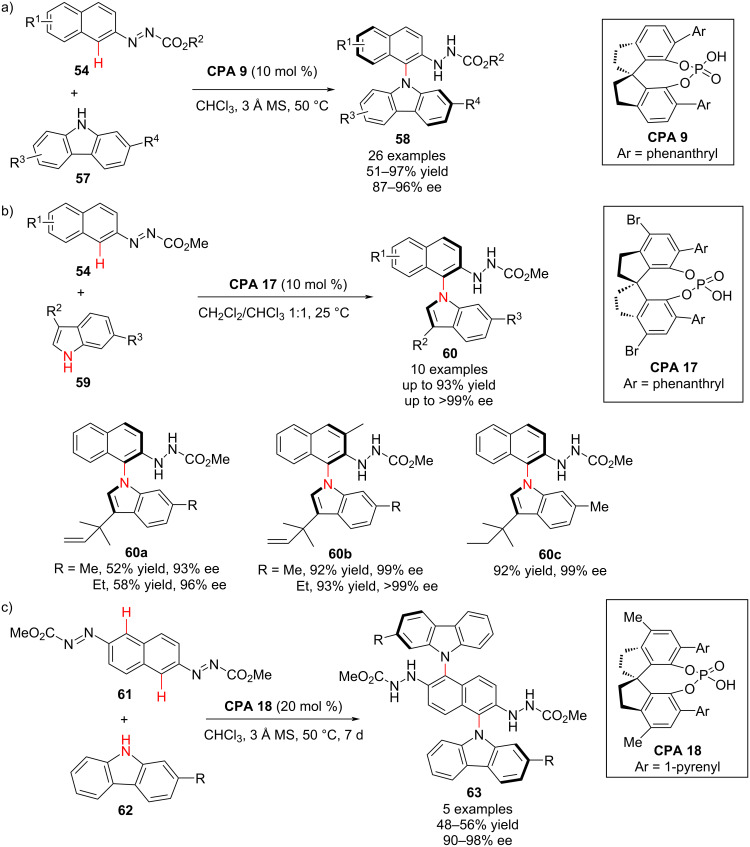
Enantioselective and atroposelective synthesis of axially chiral *N*-arylcarbazoles [73].

On the other hand, *N*-arylcarbazole frameworks are also abundant in pharmaceuticals, agrochemicals, natural products, and functional OLED materials [71–72]. Therefore, the construction of axially chiral *N*-arylcarbazoles is desirable. In this sense, Tan and co-workers (2020) developed the first chiral phosphoric acid-catalyzed atroposelective C–H amination of arenes for the synthesis of *N*-arylcarbazole structures. The atroposelective *N*-arylcarbazoles **58** were prepared by C–H amination of azonaphthalene derivatives **54** and carbazole derivatives **57** via asymmetric addition and chirality transfer. In the presence of **CPA 9**, axially chiral *N***-**arylcarbazoles were obtained in moderate to good yields (51–97%) with good to excellent enatioselectivity (87–96% ee, Scheme 20a) [73].

More importantly, the same group reported the synthesis of axially chiral *N*-arylindole atropisomers from azonaphthalene **54** and indole substrates **59** in the presence of chiral phosphoric acid **CPA 17**. The atropisomeric adducts were obtained in moderate to good yields (up to 93%) with good to excellent enantioselectivity (up to >99% ee, Scheme 20b). Therefore, this synthetic method (nucleophilic aromatic substitution reaction) is crucial for the development of alternative C–N bond-forming reactions to conventional metal-involved cross-couplings, providing axially chiral *N*-arylcarbazoles **60** in good yields with remarkable enantiocontrol through a rearomatization-enabled central to axial chirality transfer pathway [73].

Citing the crucial role of di-carbazole-substituted arenes in OLED materials [74], Tan and co-workers have recently developed structural motifs with two chiral *N*-aryl axes from 2,6-diazonaphthalene **61** and carbazoles **62**. For this reaction, a high loading of 20 mol % **CPA 18** with a 1-pyrenyl group at the 6,6’-position of the spiro backbone was preferred as the best catalyst for this transformation. Encouragingly, the double atroposelective C–H amination reaction took place and afforded the desired 1,5-dicarbazole naphthalene derivative **63** in moderate yield with >90% enantioselectivity (Scheme 20c) and tolerable diastereoselectivity (4:1) [73].

**Scheme 21:**
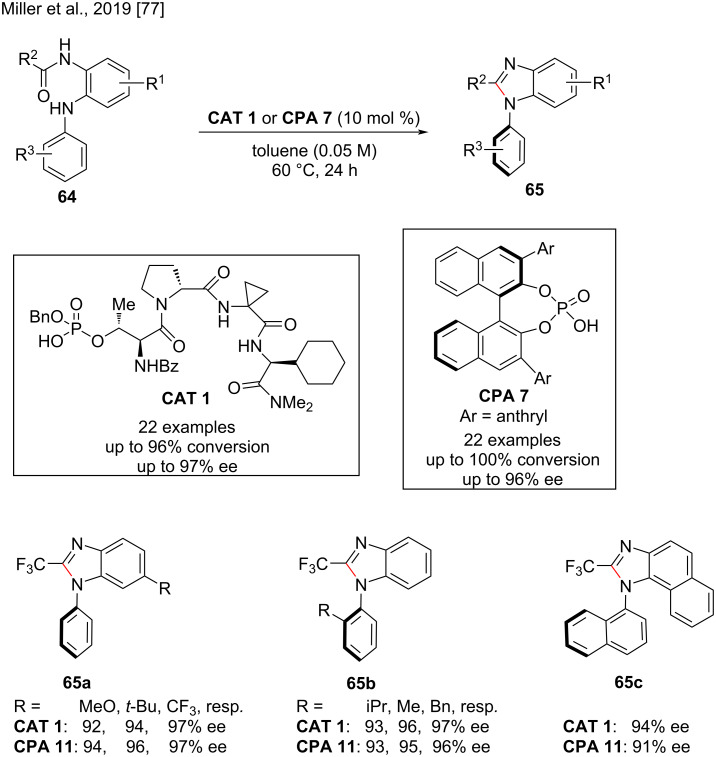
Atroposelective cyclodehydration reaction.

The optically enriched benzimidazoles are N-heterocycles which are of great interest as drug-like molecules [75], and exhibit biological activities such as anticancer, antiviral, antifungal, and antibacterial effects [76]. In this context, Miller and co-workers reported the performance of *C*_2_-symmetric chiral phosphoric acids (C_2_-type) and phosphothreonine-embedded, peptidic phosphoric acids (pThr-type CPAs) to catalyze a wide range of atroposelective cyclodehydration reactions for the synthesis of benzimidazoles. In the presence of **CPA 7**, the corresponding products **65** were obtained with the highest selectivity (up to 96% ee) at full conversion while catalyst **CAT 1** (Scheme 21) afforded **65** with up to 94% ee at 96% conversion. The highly substituted axially chiral benzimidazoles **65** were all formed with excellent enantioselectivity when either **CAT 1** (93–97% ee) or **CPA 7** (93–96% ee) was used as catalyst (Scheme 21). The authors described that, BINOL-derived CPAs and pThr-type CPA scaffolds were found to be effective for atroposelective cyclodehydrations. Both the DFT and catalyst correlation studies showed that the steric repulsion between the large 3,3′-substituent of the C2-type CPAs catalyst and the bottom aromatic ring of the substrate seems to determine the enantioselectivity [77].

**Scheme 22:**
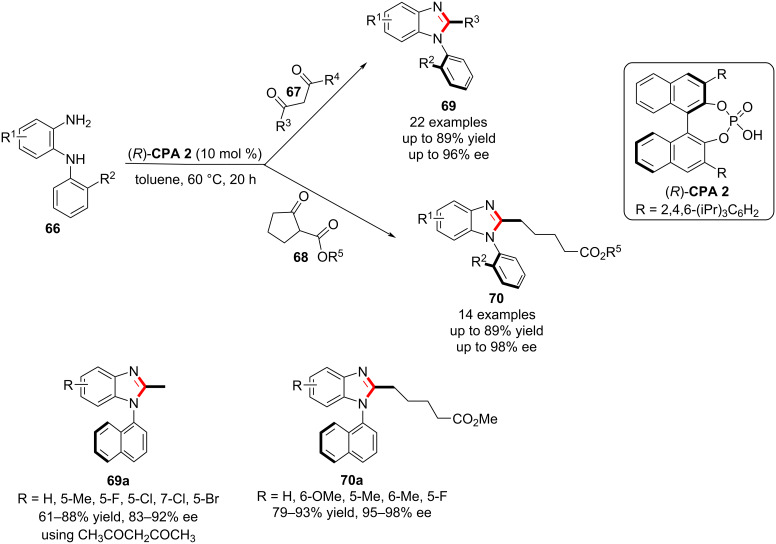
Atroposelective construction of axially chiral *N*-arylbenzimidazoles [78].

**Scheme 23:**
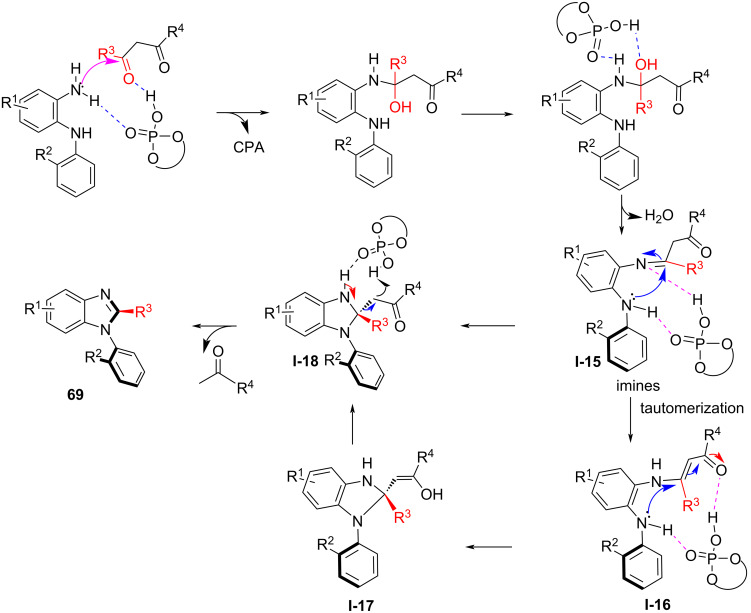
Proposed reaction mechanism for the atroposelective synthesis of axially chiral *N*-arylbenzimidazoles.

In 2020, Fu and co-workers described the chiral phosphoric acid-catalyzed atroposelective construction of axially chiral *N*-arylbenzimidazoles involving a carbon–carbon bond cleavage under optimal reaction conditions. In the presence of **CPA 2**, *N*^1^-(aryl)benzene-1,2-diamines **66** were used in the reaction with multicarbonyl compounds **67** and **68** and afforded the corresponding products, axially chiral *N*-arylbenzimidazoles **69** and **70** in high yields (up to 89%) with excellent enantioselectivity (up to 98% ee, Scheme 22) [78]. The primary amino group in the N^1^-(aryl)benzene-1,2-diamines reacts with the carbonyl group in **67** to give imine intermediate **I-15** mediated by the chiral phosphoric acid. Meanwhile, isomerization of **I-15** leads to enamine **I-16**. In the presence of the CPA catalyst, an intramolecular Michael addition of the amino group to the enamine in **I-16** leads to **I-17**. Subsequently, the imines **I-15** and **I-17** are converted to the intermediate **I-18**, which through elimination by cleavage of the C–C bond gives the target product **69** (Scheme 23). This catalytic approach encompasses a wide range of substrates and functional groups to construct atropisomeric *N*-arylbenzimidazoles that are widely used in natural products, biologically active molecules, ligands, and catalysts.

**Scheme 24:**
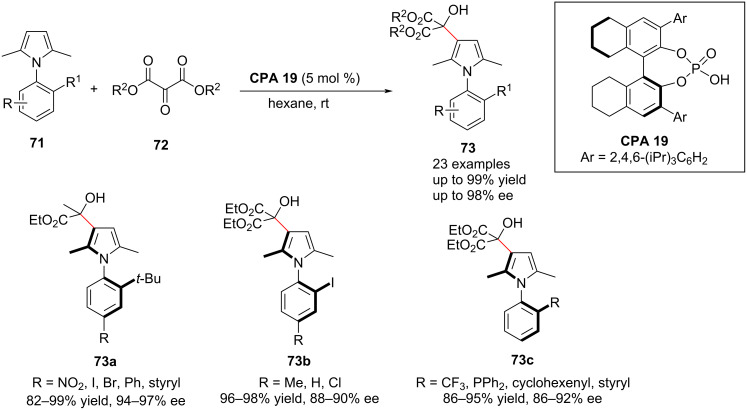
Atroposelective synthesis of axially chiral arylpyrroles [21].

Axially chiral arylpyrroles are the core scaffold for a variety of natural products, bioactive compounds and pharmacological agents [79] as well as for a variety of chiral phosphine ligands [80]. As a result, the preparation of axially chiral arylpyrroles has been one of the most important areas of investigation in synthetic chemistry. In the last decade, the optical activity of arylpyrroles has been explored by optical resolution of racemates using chiral resolving agents or chiral column chromatography [81]. Although Zhang et al. developed the first catalytic asymmetric Paal–Knorr reaction for accessing highly enantioenriched axially chiral arylpyrroles in 2017 [82], a complicated catalytic system and narrow substrate range limited its application. Therefore, the use of chiral phosphoric acids to modulate axial chirality enables the preparation of highly enantioenriched axially chiral arylpyrroles. In this context, Tan and co-workers discovered in 2019 a highly effective approach using organocatalytic atroposelective desymmetrization and kinetic resolution to obtain enantioenriched axially chiral arylpyrroles. The axially chiral arylpyrroles **73** were prepared in high yields (up to 99%) and with excellent enantioselectivities (up to 98% ee) from the reaction of **71** and diethyl ketomalonate (**72**) under remote control of **CPA 19** as chiral phosphoric acid catalyst (Scheme 24). The hydrogen bonding between the ketomalonate and **CPA 19** proved to be the most important interaction in the formation of the chiral pocket for the induction of chirality and could considerably improve the stereocontrol of the reaction [21].

**Scheme 25:**
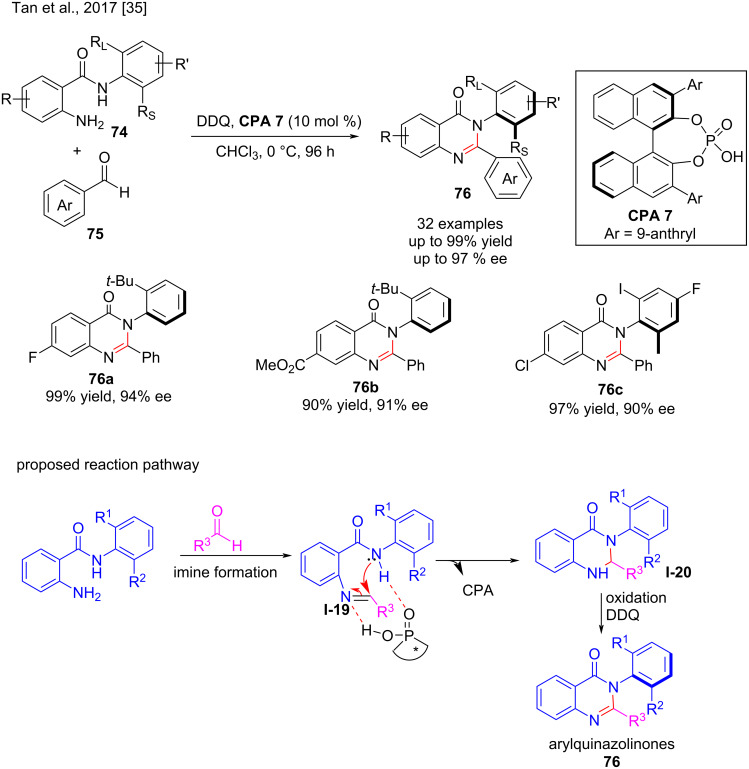
Synthesis of axially chiral arylquinazolinones and its reaction pathway [35].

The axially chiral arylquinazolinones form the backbones of a large number of natural products and biologically active compounds as well as chiral ligands [83]. Nevertheless, a simple chiral phosphoric acid-catalyzed enantioselective approach to access optically pure arylquinazolinones has never been developed in the last decades. However, in 2017, Tan and co-workers reported the enantioselective synthesis of axially chiral arylquinazolinones **76** from the reaction of *N*-arylanthranilamides **74** and benzaldehyde (**75**) by accelerating imine formation (**I-19**), and under the catalysis of a chiral phosphoric acid, intramolecular nucleophilic addition occurs to form **I-20**, followed by oxidative dehydrogenation with 2,3-dichloro-5,6-dicyano-1,4-benzoquinone (DDQ). In the presence of 10 mol % chiral phosphoric acid **CPA 7**, the axially chiral arylquinazolinones **76** were obtained in high yield (up to 99%) with high enantioselectivity (83–97% ee, Scheme 25). Both the position and the electronic properties of the substituents on the aromatic ring had a minor influence on the reaction efficiency and enantioselectivity of this transformation [35].

**Scheme 26:**
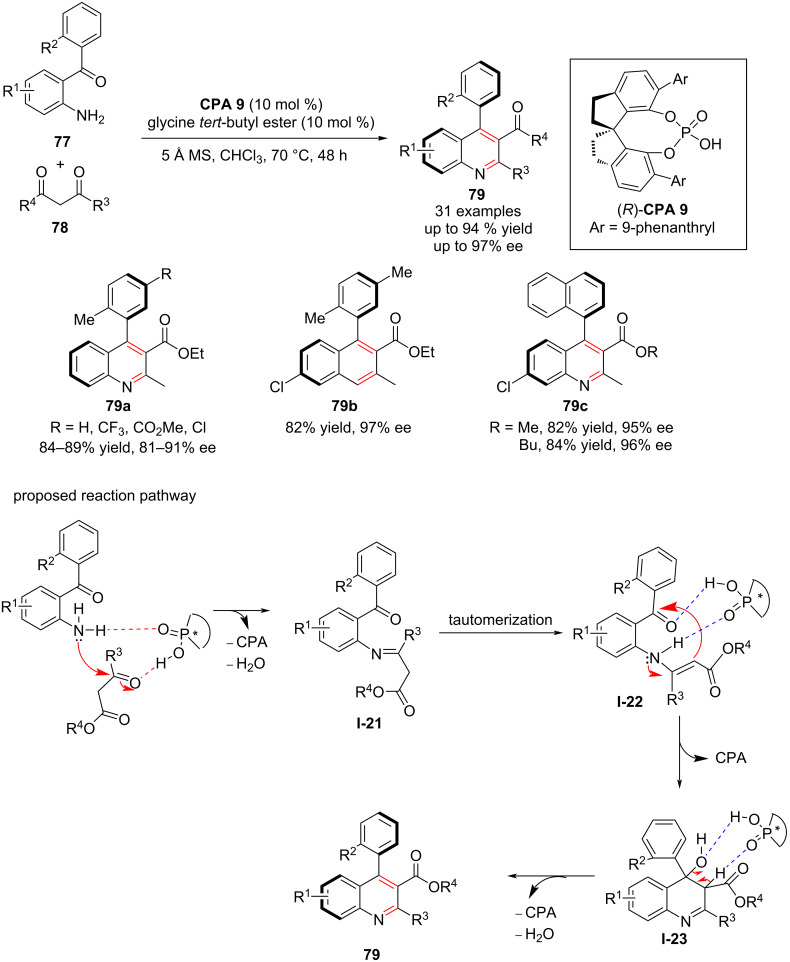
Synthesis of axially chiral aryquinoline by Friedländer heteroannulation reaction and its proposed reaction mechanism [85].

Quinolines are widely used in natural and synthetic products and exhibit remarkable pharmacological properties [84]. To synthesize this valuable scaffold, Cheng and co-workers performed for the first time in 2019 an atroposelective Friedländer heteroannulation reaction of 2-aminoaryl ketones **77** with α-methylene carbonyl derivatives **78** catalyzed by a chiral phosphoric acid. In the presence of (*R*)-**CPA 9**, the Friedländer heteroannulation reaction between aromatic amines **77** and the carbonyl derivative **78** was carried out to form imine **I-21**, which tautomerized to generate intermediate **I-22**. Under **CPA 9** catalysis, the intermediate **I-22** is converted to **I-23**, which after dehydration forms the desired enantioenriched products, polysubstituted 4-arylquinolines **79** in high yield (up to 94%) with good to excellent enantioselectivities (up to 97% ee, Scheme 26). The chiral phosphoric acid catalyst played an important role in the asymmetric induction by establishing a favorable chiral environment in the cyclization step through supporting hydrogen bonds [85].

**Scheme 27:**
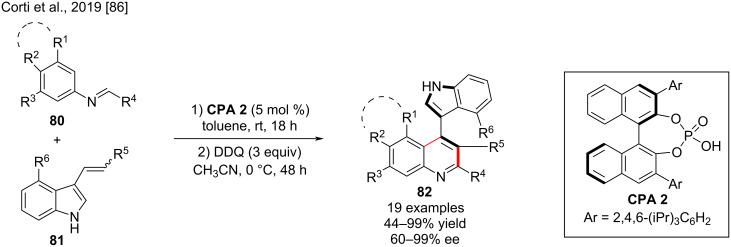
Povarov cycloaddition–oxidative chirality conversion process.

In 2019, the group of Bertuzzi and Corti developed chiral phosphoric acid-catalyzed Povarov reactions of *N*-arylimines **80** with 3-alkenylindoles **81** to give enantioenriched, highly substituted, 1,2,3,4-tetrahydroquinolines, which can be oxidized to axially chiral 4-(indol-3-yl)quinolones **82**, in a central-to-axial chirality conversion approach with up to 99% yield and high enantioselectivity (up to 99% ee, Scheme 27). In presence of 5 mol % (*R*)-**CPA 2**, the benzyl-substituted 3-alkenylindole **81** (dienophile) was subjected to a Povarov cycloaddition with the commonly used imine **80**, giving the 2,3,4-tetrahydroquinoline as a single diastereomer in high yield and enantioselectivity and finely tolerating the high steric requirements necessary to provide stable atropisomeric quinolines after oxidation by DDQ [86].

### 3. Enantioselective synthesis of axially chiral arylalkene and *N*-arylamines

**Scheme 28:**
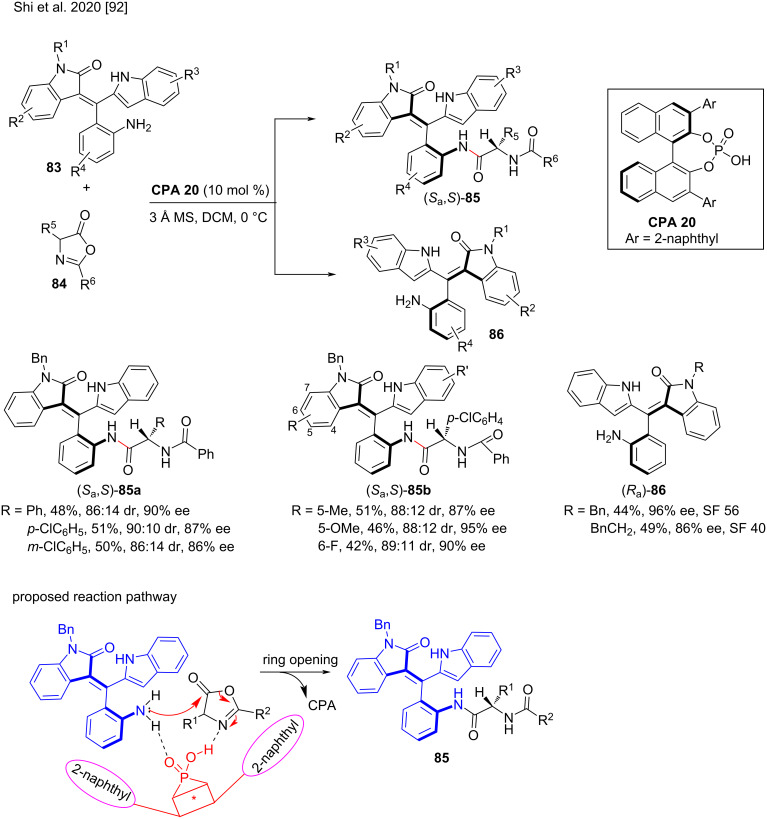
Atroposelective synthesis of oxindole-based axially chiral styrenes via kinetic resolution.

Although many elegant strategies have been developed to enable the atroposelective construction of axially chiral biaryls and heterobiaryls [87–89], the synthesis of axially chiral styrenes or vinylarenes has rarely been developed [90–91], due to their low rotational barrier and weak configurational stability. Moreover, the application of an organocatalytic kinetic resolution strategy to access axially chiral styrenes has rarely been reported. In this regard, Shi and co-workers reported the first atroposelective access to oxindole-based axial chiral styrenes or vinylarenes by kinetic resolution of racemic oxindole-based styrenes **83** and azalactones **84**. In the presence of **CPA 20**, racemic oxindole-based styrenes reacted with azalactones via hydrogen bonding, and the azalactones underwent asymmetric ring opening to form axially chiral oxindole-based styrenes **85** in up to 53% yield with good diastereoselectivities (up to 94:6 dr) and enatioselectivities (up to 95% ee)**.** Moreover, the racemic oxindole-based styrenes underwent kinetic resolution to afford other axially chiral styrenes **86** in moderate yields (up to 54%) with excellent enantioselectivities (up to 96% ee) and high selectivity factors (SF up to 106) [92]. This strategy is critical for accessing axially chiral the oxindole-based styrenes and provides a robust method for the synthesis of bisamide derivatives that are both axially and centrally chiral (Scheme 28).

**Scheme 29:**
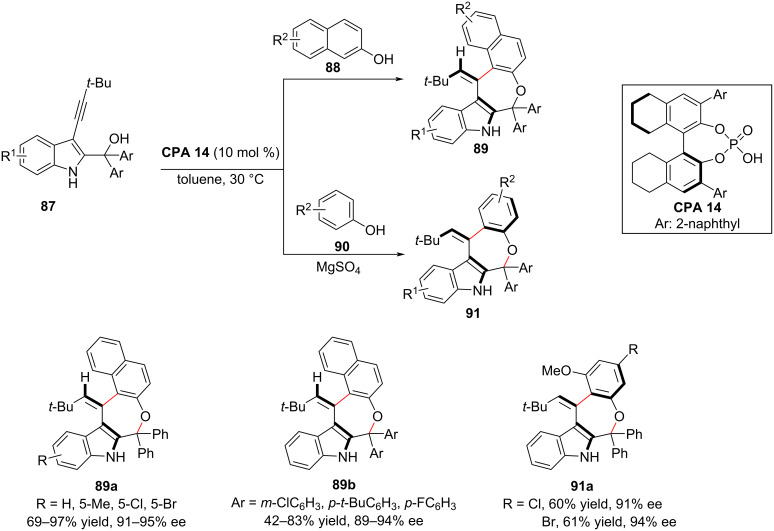
Synthesis of axially chiral alkene-indole frame works [45].

**Scheme 30:**
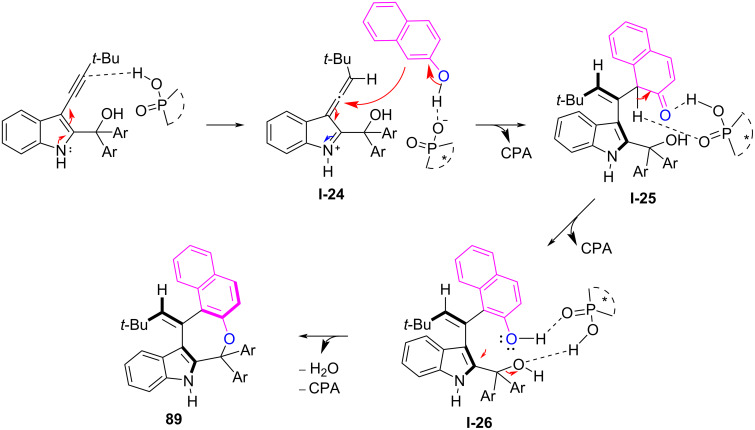
Proposed reaction mechanism for axially chiral alkene-indoles.

Very recently, the same group reported the first CPA-catalyzed asymmetric assembly of axially chiral arylalkene-indole scaffolds by organocatalytic (*Z*/*E*)-selective and enantioselective (4 + 3)-cyclization using (3-alkynylindol-2-yl)methanols **87** as electrophile and 2-naphthols **88** or phenols **90** as nucleophile (Scheme 29) [45]. The (3-alkynylindol-2-yl)methanol **87** is expected to convert to the allene-iminium intermediate **I-24** by accepting a proton from **CPA 14**. Then, the CPA anion activates the nucleophilic addition between 2-naphthol (**88**) and allene-iminium intermediate **I-24** to form axially chiral **I-25**, followed by rearomatization of the naphthol ring of **I-25** and isomerization to **I-26**. Thereafter, CPA forms two hydrogen bonds with the two OH groups of **I-26** to generate a carbocation and facilitates an intramolecular nucleophilic addition to afford axially chiral aryl-alkene-indole frameworks (**89**) in up to 98% yield, >95:5 *E*/*Z*, and up to 97% enantioselectivity (Scheme 30).

**Scheme 31:**
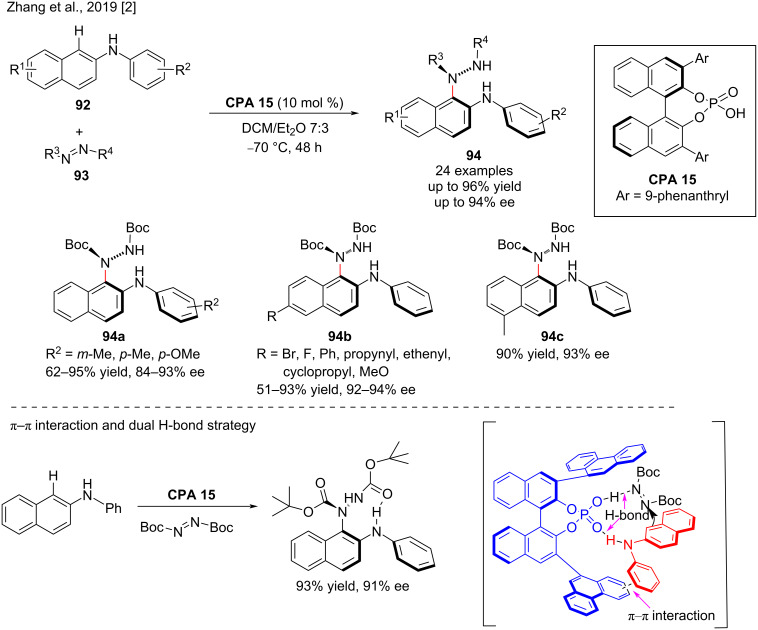
Atroposelective C–H aminations of *N*-aryl-2-naphthylamines with azodicarboxylates.

The nonbiaryl N–C atropisomer is an important structural scaffold, which is present in natural products, medicines. and chiral ligands due to the restricted rotation around an N–C single bond. There are few strategies for the catalytic atroposelective construction of N–C nonbiaryl atropisomers, which mainly consist of enantioselective cyclization [35], N-functionalization [93], and desymmetrization [94] of the existing achiral N–C bond. In this context, Bai and co-workers (2019) developed the direct intermolecular enantioselective C–H amination of *N*-aryl-2-naphthylamines **92** with azodicarboxylates **93** to prepare N–C atropisomers of nonbiaryl naphthalene-1,2-diamine **94**. In the presence of chiral phosphoric acids (**CPA 15**), the desired product **94** was obtained in moderate to high isolated yield (up to 95%) and enantioselectivity (up to 94% ee, Scheme 31). However, the reactivity and stereoselectivity decreased the closer the substituents were to the reaction site, probably due to steric effects. On the other hand, the substrates with electron-withdrawing groups would decrease the electron cloud density, limiting the reactivity and thus reducing the yield. In general, the electronic properties of the substituents did not affect the stereoselectivity of the reaction. The mechanistic study revealed that, **CPA 15** simultaneously activates *N*-phenyl-2-naphthylamine and azodicarboxylate through a dual hydrogen bond activation mode and a π–π interaction strategy (Scheme 31) [2].

**Scheme 32:**
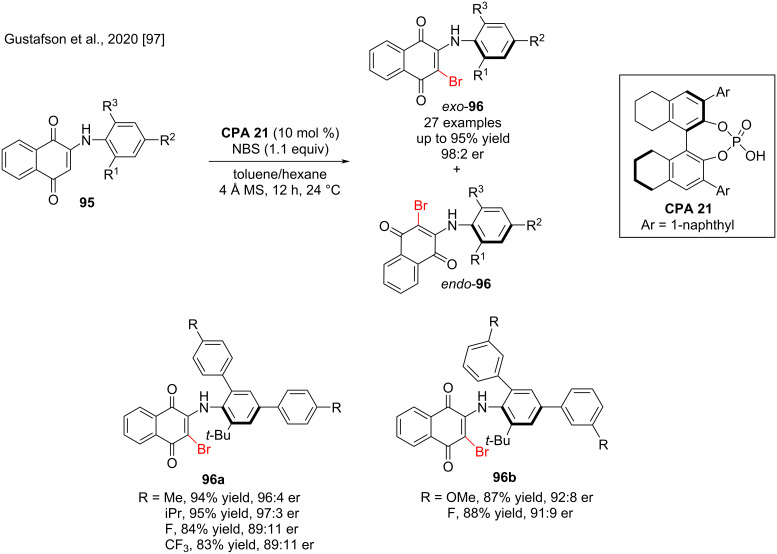
Synthesis of brominated atropisomeric *N*-arylquinoids.

Diarylamines and related scaffolds are among the most common potentially atropisomeric chemotypes in medicinal chemistry. For example, the drugs binimetinib and bosutinib contain diarylamines that exist as rapidly interconverting atropisomers, and a VEGFR inhibitor from Wyeth contains a potentially atropisomeric *N*-arylquinoid [95–96]. In 2020, Gustafson and co-workers reported the first chiral phosphoric acid-catalyzed atroposelective electrophilic halogenation of *N*-arylquinoids **95**, a class of compounds similar to diarylamines. In this reaction, **CPA 21** was an effective catalyst, providing stereochemically stable brominated product *N*-arylquinoids **96** in high yield (up to 95%) and atroposelectivity (up to 98:2 er, Scheme 32). Remarkably, these products existed as stereochemically stable class 3 atropisomers under protic and aprotic conditions due to a strong intramolecular N–H–O hydrogen bond that locks one of the axes into a planar conformation and proposed that the quinoid-nitrogen axis exists in a planar *exo* conformation [97].

### 4. Enantioselective synthesis of axially chiral allenes and spiranes

The enantioselective construction of spirocyclic centers is an exciting synthetic challenge that plays a prominent role in the discovery of new catalysts and complex molecules [98]. Spirocyclic frameworks are present, for example, in biologically active natural products (e.g., fredericamycin A and acutumine [99]), SPINOL-based ligands and catalysts [100], and organometallic complexes with important applications. The use of chiral catalysts for spirocyclization reactions brings asymmetry at the site of the spirocyclic center while tolerating different electronic and steric functional groups [98].

**Scheme 33:**
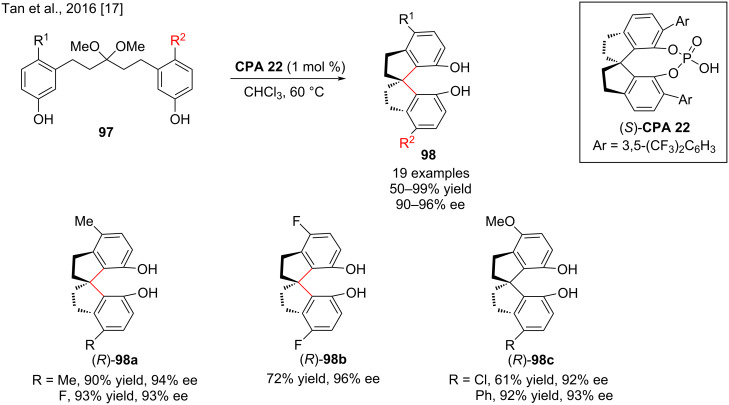
The enantioselective syntheses of axially chiral SPINOL derivatives.

In this context, Tan and co-workers succeeded in the enantioselective synthesis of axially chiral SPINOLs **98** from ketals **97** by an intramolecular fashion in the presence of 1 mol % chiral phosphoric acid **CPA 22** to afford the axially chiral product (*R*)-**98** in high yield (62–95%) with good to excellent enantioselectivity (90–96% ee, Scheme 33) [17]. The electronic properties and steric bulk of the substituents on the catalysts as well as the axial chiral backbone have a very strong influence on the reactivity and enantioselectivity as shown in Scheme 33. The synthesis of the products (*R*)-**98** at a gram scale was carried out without loss of chemical yields and stereoselectivities under optimized conditions. The authors also found that the excellent stereocontrol was due to the simultaneous interaction between the bifunctional phosphoric acid and the intermediate formed in the reaction via hydrogen bonds.

**Scheme 34:**
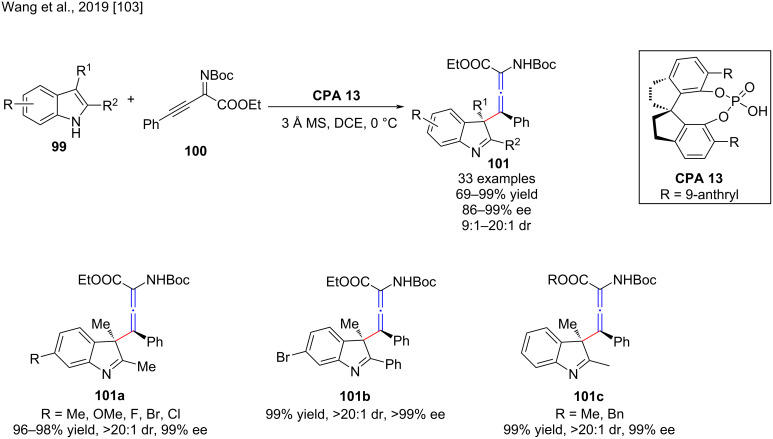
γ-Addition reaction of various 2,3-disubstituted indoles to β,γ-alkynyl-α-imino esters.

Axially chiral allenes and derivatives occur in overwhelming numbers in natural products, pharmaceuticals, functional materials, and useful intermediates in organic synthesis [101]. Therefore, great efforts have been made to synthesize these axially chiral scaffolds [102]. In this regard, Wang and co-workers reported the organocatalytic asymmetric synthesis of tetrasubstituted α-amino allenoates by a dearomative γ-addition reaction of 2,3-disubstituted indoles **99** to β,γ-alkynyl-α-imino esters **100** [103]. In the presence of chiral phosphoric acid **CPA 13**, a series of tetrasubstituted α-amino allenoates **101** was prepared in moderate to excellent yields (69–99%), dr (9:1 to >20:1), and excellent enantioselectivity (91–99% ee, Scheme 34). The mechanistic studies showed that the substituents at the second and third positions of the indole play a crucial role in the chemo- and stereoselectivity. Moreover, the chiral phosphoric acid group attains a bifunctional role by activating both partners via hydrogen bonds, and the chiral backbone of the catalyst controls the stereoselectivity through steric effects and π–π interactions.

**Scheme 35:**
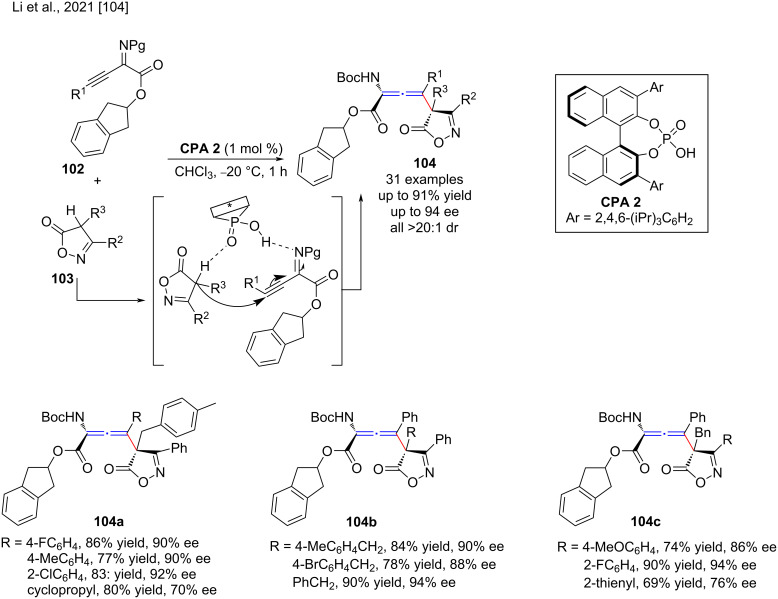
Regio- and stereoselective γ-addition reactions of isoxazol-5(4*H*)-ones to β,γ-alkynyl-α-imino esters.

Moreover, Li and co-workers recently developed CPA-catalyzed regio- and stereoselective γ-addition reaction of isoxazol-5(4*H*)-ones **103** to β,γ-alkynyl-α-imino esters **102** for the synthesis of axially chiral tetrasubstituted α-amino allenoates **104** containing an adjacent quaternary carbon stereocenter and an axially chiral tetrasubstituted allene scaffold [104]. Although isoxazol-5(4*H*)-ones have different nucleophilic sites, the authors succeeded in the C-allenylation of isoxazol-5(4*H*)-ones with high efficiency (up to 91% yield) and high regioselectivities and stereoselectivities (up to 94% ee and >20:1 dr, Scheme 35).

**Scheme 36:**
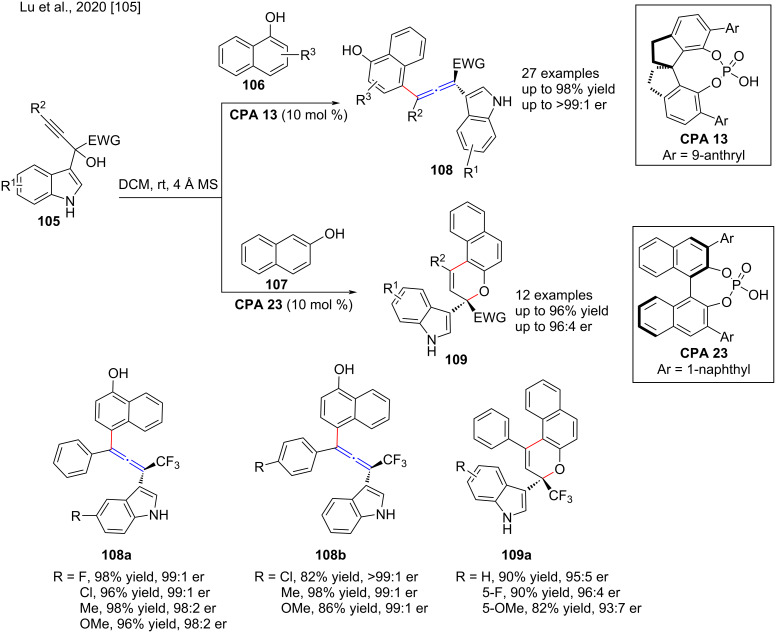
Synthesis of chiral tetrasubstituted allenes and naphthopyrans.

Because of the importance of chiral tetrasubstituted allenes with aryl substituents, the asymmetric synthesis of these scaffolds has received much attention. In 2020, Lu and co-workers carried out the enantioselective dehydrative γ-arylation of α-indolyl-α-trifluoromethylpropargyl alcohol **105** and 1-naphthol (**106**) or 2-naphthol (**107**) catalyzed by chiral phosphoric acids **CPA 13** and **CPA 23**, to produce a wide range of chiral tetrasubstituted allenes **108** and naphthopyrans **109** in high yield (≤98% yield) with excellent regio- and enantioselectivities (>99:1 er, Scheme 36) [105]. However, when substituents are present at the C-4 or C-7 position of the indole ring of the propargyl alcohol, the yield decreases, which could be due to steric effects. Since the chiral phosphoric acid catalysts can interact with these groups via double hydrogen bonds, control studies have shown that the free OH on naphthol/phenol and the NH groups on the α-indolyl-α-trifluoromethylpropargyl alcohol are very important for the reaction.

**Scheme 37:**
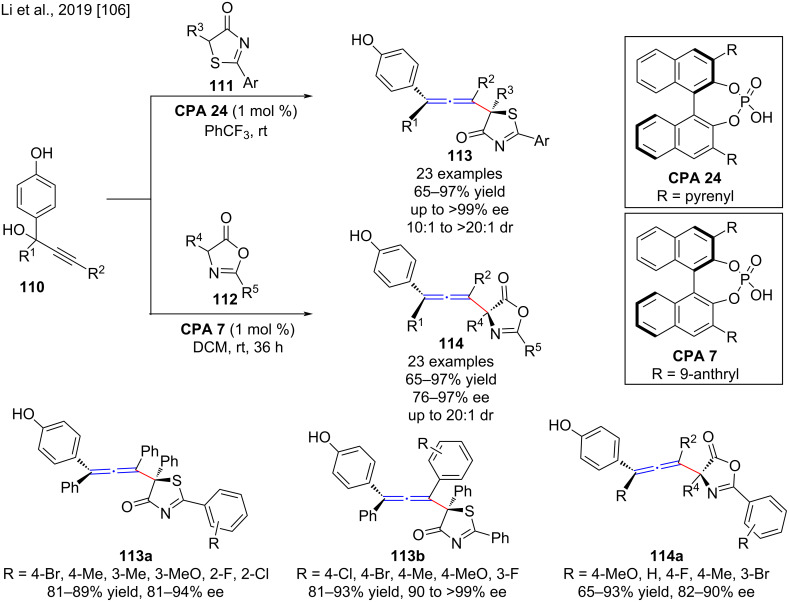
Asymmetric remote 1,8-conjugate additions of thiazolones and azlactones to propargyl alcohols.

Li and co-workers developed a chiral phosphoric acid-catalyzed asymmetric remote 1,8-conjugate addition of thiazolones **111** and azlactones **112** to propargyl alcohols **110** for the synthesis of the chiral allenes **113** and **114**, respectively. In the presence of 1 mol % **CPA 24**, 5*H*-thiazol-4-ones **111** and *p-*quinone methides generated in situ from propargyl alcohols **110** were incorporated and afforded the axially chiral tetrasubstituted allenes **113** with a chiral thiazolone moiety in high yield (65–97%), high enantioselectivity (76 to *>*99% ee) and diastereoselectivity (10:1 to *>*20:1 dr). In addition, the enantioselective 1,8-conjugate addition of azlactones **112** to para-quinone methides generated in situ from propargyl alcohols **110** were carried out in the presence of 1 mol % chiral phosphoric acid **CPA 7** and afforded the chiral allenes **114** in high yields (65–97%) with good to excellent enantioselectivities (76–97% ee) and good diastereoselectivities (up to 20:1 dr, Scheme 37) [106]. The electronic nature and position of the substituents on the aromatic ring of the thiazolones or propargylic alcohols did not significantly affect the stereoselectivity of the reaction. Moreover, both electron-withdrawing and donating groups on the aromatic rings of propargyl alcohols or azlactones smoothly participated in the asymmetric 1,8-conjugate addition and afforded the corresponding chiral allenes in good yields with high enantioselectivity. The control experiments showed that the propargyl alcohol **110** was firstly converted to *para*-quinone methide (*p*-QM) in the presence of CPA and then led to the successful formation of the corresponding products through hydrogen-bonding interaction of *para*-quinone methide and thiazolones/azlactones with CPA.

**Scheme 38:**
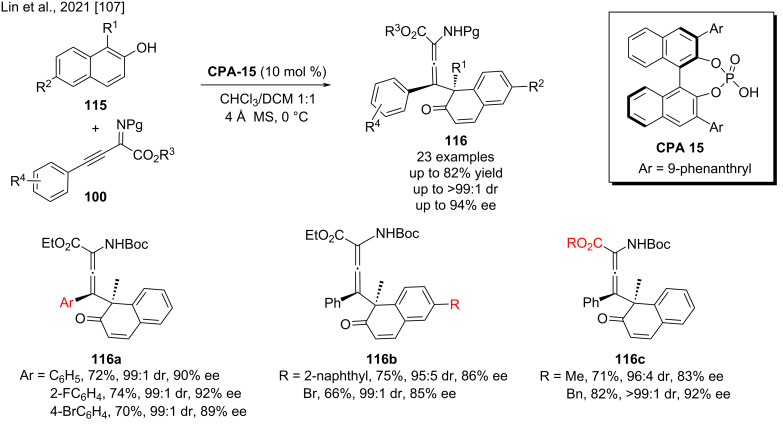
Synthesis of chiral allenes from 1-substituted 2-naphthols [107].

Recently, using chiral phosphoric acid catalysis, we developed a highly regio-, diastereo-, and enantioselective dearomatization reaction of 1-substituted 2-naphthols **115** and β,γ-alkynyl-α-imino esters **100**. The highly functionalized naphthalenone derivatives **116** with an allene moiety, exhibiting both a quaternary stereocenter and axial chirality, were obtained in good yields (up to 82%) with high diastereoselectivities (up to >99:1 dr), and enantioselectivities (up to 96% ee, Scheme 38). Control experiments showed that the high to excellent stereoselectivity is the result of a dual hydrogen-bonding interaction between the substrates and the chiral phosphoric acid, with the substituent at the 1-position of the 2-naphthol playing a key role in controlling the regioselectivity [107].

## Conclusion

In recent decades, chiral phosphoric acids have been recognized as privileged chiral catalysts and ligands and have therefore become an important tool in asymmetric organic synthesis. This review summarizes the state of the art in the chiral phosphoric acid-catalyzed asymmetric synthesis of axially chiral biaryls, heterobiaryls, arylamines, arylalkenes or styrenes, spiranes, and allenes. These axially chiral compounds have attracted considerable attention in recent years due to their wide application in the total synthesis of axially chiral natural products, functional materials, bioactive compounds, privileged chiral ligands, and have further potential applications in asymmetric catalysis and drug discovery. Accordingly, considerable efforts have been made to find new efficient routes for the enantioselective construction of various atropisomeric aryl–aryl or aryl–heteroaryl, enantioselective spiranes and allenes. Despite the advances mentioned above, much of this area of research is still relatively unexplored. Compared to the use of chiral phosphoric acids in the preparation of centrally chiral compounds, their application in the synthesis of axially chiral biaryls and heterobiaryls, axially chiral allene, atropisomeric aryl alkenes, and spirane moieties is still quite limited. Given the increasing importance of axially chiral compounds, novel chiral phosphoric acid-catalyzed asymmetric methods to address these challenges are highly desirable. Therefore, we believe that the future development of asymmetric syntheses using chiral phosphoric acid catalysts will play a crucial role in the preparation of other complex organic molecules with axial chirality, which will be widely used in science and industry in the near future.
